# Repurposing Metformin for periodontal disease management as a form of oral-systemic preventive medicine

**DOI:** 10.1186/s12967-023-04456-1

**Published:** 2023-10-10

**Authors:** Vitor C. M. Neves, Luciana Satie Okajima, Eyad Elbahtety, Susan Joseph, James Daly, Athul Menon, Di Fan, Ayste Volkyte, Giuseppe Mainas, Kathy Fung, Pawan Dhami, Andre A. Pelegrine, Paul Sharpe, Luigi Nibali, Mark Ide

**Affiliations:** 1 Centre for Craniofacial and Regenerative Biology, FoDOCS, King’s College London, London, UK; 2 Periodontology Unit, Centre for Host-Microbiome Interactions, FoDOCS, King’s College London, London, UK; 3 Department of Periodontology and Implantology, School of Dentistry, São Leopoldo Mandic, Campinas, Brazil; 4 NIHR BRC Genomics Research Platform, Guy’s and St Thomas’ NHS Foundation Trust, King’s College London School of Medicine, London, UK; 5 Institute of Animal Physiology and Genetics, Brno, Czech Republic

## Abstract

**Background:**

Despite the improvements in treatment over the last decades, periodontal disease (PD) affects millions of people around the world and the only treatment available is based on controlling microbial load. Diabetes is known to increase the risk of PD establishment and progression, and recently, glucose metabolism modulation by pharmaceutical or dietarian means has been emphasised as a significant modulator of non-communicable disease development.

**Methods:**

The impact of pharmaceutically controlling glucose metabolism in non-diabetic animals and humans (REBEC, UTN code: U1111-1276-1942) was investigated by repurposing Metformin, as a mean to manage periodontal disease and its associated systemic risk factors.

**Results:**

We found that glucose metabolism control via use of Metformin aimed at PD management resulted in significant prevention of bone loss during induced periodontal disease and age-related bone loss in vivo. Metformin also influenced the bacterial species present in the oral environment and impacted the metabolic epithelial and stromal responses to bacterial dysbiosis at a single cell level. Systemically, Metformin controlled blood glucose levels and age-related weight gain when used long-term. Translationally, our pilot randomized control trial indicated that systemic Metformin was safe to use in non-diabetic patients and affected the periodontal tissues. During the medication window, patients showed stable levels of systemic blood glucose, lower circulating hsCRP and lower insulin levels after periodontal treatment when compared to placebo. Finally, patients treated with Metformin had improved periodontal parameters when compared to placebo treated patients.

**Conclusion:**

This is the first study to demonstrate that systemic interventions using Metformin in non-diabetic individuals aimed at PD prevention have oral-systemic effects constituting a possible novel form of preventive medicine for oral-systemic disease management.

**Supplementary Information:**

The online version contains supplementary material available at 10.1186/s12967-023-04456-1.

## Introduction

Periodontal disease (PD) is a multifactorial inflammatory disease associated with microbial dysbiosis that requires cell turnover and inflammatory regulation for coping with the natural bacterial aggression against the supporting tissues of the tooth [1, 2]. The gingival tissues suffer continuous bacterial challenge over one’s lifespan, and the day-to-day treatment and prevention for PD relies solely on antimicrobial management (transient removal of plaque and use of adjunct antibiotic therapy) [3–5]. Despite the improvements in treating PD over the decades, gingivitis affects 90% of the global population and periodontitis has still been shown to be the sixth most common disease affecting mankind [6]. Given the high prevalence and preventable nature of this disease, there is great need for development of a more effective, easy to use, and minimally invasive management pathway to control PD establishment and progression without relying on anti-microbial therapy.

The biofilm-driven PD treatments currently offered in day-to-day practices do not directly address the host’s metabolic-mediated inflammatory response driving PD progression. Several metabolic factors can affect the inflammatory response, tissue healing, and immune function that are crucial for the management of periodontal disease. Among them are sugar metabolism, adipose-led inflammatory reaction, nutrition, and oxidative stress [7, 8]. Aging, a progressive inflammatory and metabolic deregulation of physiology, has been shown to be an independent risk factor for PD [9, 10]. While the metabolic changes associated with aging can vary among individuals, some common trends are observed, such as decline of Basal Metabolic Rate [11, 12], decline in the production of hormones, e.g. insulin-like growth factor 1 (IGF-1) [13], reduced mitochondrial function leading to replication errors [14], decline in insulin sensitivity, leading to insulin resistance, and alterations in lipid metabolism [15, 16]. So far, treatments for PD do not address any of these metabolic factors associated with the disease progression, therefore refreshing novel approaches to manage systemic health alongside oral health can help prevent oral-systemic disease progression.

It is estimated that approximately 50% of the adult population over 30 years have some form of PD, and this figure increases to 70% in adults over 65 years and older [17, 18]. Glucose metabolism (GM) control by pharmaceutical or dietarian means has been emphasized as a significant modulator of longevity, decreasing the development of non-communicable diseases [19]. Although the effects of longitudinal GM control on periodontal disease (PD) have never been shown in normoglycemic individuals, it is widely known that patients with poorly controlled diabetes are at a greater risk of developing PD. Further, patients successfully treated for PD have improvements in their GM, positively affecting cardiovascular health [20, 21].

Glucose metabolism has been shown to orchestrate the behaviour of inflammatory cells [22], stem cell proliferation, and lineage commitment [23], directly impacting the development of non-communicable diseases. Integrating GM and inflammatory modulation in the prevention and treatment of PD could potentially indirectly address multiple overlapping risk factors of oral and systemic diseases. Numerous glucose metabolism modulators have been investigated through both novel and re-purposed drugs [24, 25]. Here, we investigated the effects of Metformin as a pharmaceutical glucose metabolism modulator, as it is a cost-effective, safe and easy to repurpose medication, currently used as a first-line agent for glycaemic control [26]. Through directly targeting of systemic GM and knock-on effects in inflammation, Metformin has also been shown to promote longevity via control of non-communicable diseases [27, 28].

Systemic Metformin has been previously shown in vivo to improve bone levels in a periodontal treatment model [29], however Metformin has only been used as a local adjunct to periodontal treatment in clinical trials, demonstrating effective in improving periodontal clinical outcomes [30]. Here, we investigated the effect of systemic Metformin on PD management using a translational approach consisting of preclinical models and a pilot randomized control trial. We used preclinical models to investigate the effect of preventive use of Metformin on PD establishment and progression. Further, a pilot randomized control trial was set up to investigate the impact of the use of systemic Metformin in normoglycemic periodontal patients undergoing treatment to modulate systemic and periodontal inflammation.

## Materials and methods

### Animal work

All animals used in this study were handled in accordance with UK Home Office Regulations project license P5F0A1579 and personal license I6517C8EF, approved by the KCL animal ethics committee and comply with ARRIVE guidelines. Experimental procedures were approved by the King’s College Ethical Review Process. CD1 (Wild-type) male mice (n = 60) were obtained from the New Hunts House Biological Services Unit and were collected at 14 and 19 weeks old for the *P.gingivalis* colonisation experiment and 12 and 18 months for the aging experiment. The mice were kept in a single cage in 12-h cycles of light and dark (7am-7 pm), with the room temperature raging from 20 to 24 °C, room humidity at 50–60%, and were fed irradiated PicoLab® Rodent Diet 20 (20% protein and 4.5% fat) for the duration of the experiments.

Metformin (Merck) was administered in drinking water at 2 mg/ml ad libitum for 6 months in ageing experiments or for 11 weeks in *P.gingivalis* colonisation experiments. This dose was chosen because the optimal oral Metformin dose for many diabetic patients (approximately 20 mg/kg/day) has been translated into the mouse equivalent dose of 250 mg/kg/day and was shown successful in a longevity study [26–28].

### *P. gingivalis* colonisation experiment

*P. gingivalis* W50 was grown on blood agar plates containing 5% defibrinated horse blood or in Brain Heart Infusion broth (BHI) (Oxoid) supplemented with haemin (5 μg/mL) in an anaerobic atmosphere of 80% N_2_, 10% H_2_ and 10% CO_2_ at 37 °C (Don Whitley Scientific). Oral gavage with *P. gingivalis* was performed as described in Baker et al. [31]. Briefly, during the period of a week, mice were orally inoculated by means of a ball-ended feeding needle three times at two-day intervals with 10^9^ CFU *P. gingivalis* W50 suspended in 2% carboxy-methylcellulose (Merck) vehicle. Only one course of inoculation was performed.

### Weight and blood glucose measurement

Weekly weight and blood glucose measurements were acquired in the morning. Blood glucose measurements were done using Accu-Chek Aviva (Roche) [32] with blood drawn from the murine tail vein.

### μCT analysis

Mice maxillae and mandibles were collected, fixed in PFA 4% overnight at 4 °C, and scanned using a Bruker Skyscan1272 μCT scanner. Microview (Parallax Innovations) software programme was used for visualization and analysis. Three-dimensional (3D) reconstructions were used to verify alveolar bone loss and acquire images. Alveolar bone loss was verified using the line segment tool from the cementum-enamel junction to alveolar bone crest (CEJ-ABC) in 6 points around all 12 molars, replicating clinical 6-point pocket chart (Mesial buccal; Buccal; Distal buccal; Mesial lingual/palatal; Lingual/Palatal; Distal lingual/palatal). The reported outcomes of the vertical bone loss represent the average of all sites measured in each mouse.

### qPCR

RNA was extracted from the gingiva of the mice in the *P.gingivalis* colonisation experiment (both the inoculated and non-inoculated mice) (n = 6/group) using TRIzol (Thermo Fisher Scientific) as recommended by the manufacturer. The RNA was quantified using Nanodrop and reverse transcribed into cDNA. Beta-actin was used as housekeeping gene (Forward- GGCTGTATTCCCCTCCATCG, Reverse- CCAGTTGGTAACAATGCCTGT), Igf-1 (Forward- GGAGATGTACTGTGCCCCAC, Reverse- TAGGGACGGGGACTTCTGAG), Axin2 (Forward-TGACTCTCCTTCCAGATCCCA, Reverse-TGCCCACACTAGGCTGACA), Tnf-α (Forward- AGCCGATGGGTTGTACCTTG, Reverse- ATAGCAAATCGGCTGACGGT), and Caspase 3 (Forward- AGCTTGGAACGGTACGCTAA, Reverse- GAGTCCACTGACTTGCTCCC).

### 16 s rRNAseq

The murine oral cavity was swabbed for 30 s, using sterile fine tip rayon swabs (VWR International) after the animal was culled, and the swabs were stored in 100 µl reduced John’s transport medium at -70 °C. Whole genomic DNA was extracted from the above swabs using the DNeasy PowerSoil Pro Kit (Qiagen) according to manufacturer’s instructions. PCR reactions targeting the V1-V2 variable regions of the 16S rRNA gene were performed as previously described in Joseph et al. [33]. Extraction kit controls and PCR negative controls were included in the amplification plates as well as sequencing pools. Pooled amplicons were sequenced at the Barts and the London Genome Centre using an Illumina MiSeq 2 × 250 flow cell for paired-end sequencing.

The generated reads were quality checked, filtered, trimmed, denoised, dereplicated and assembled into amplicon sequence variants (ASVs) using the DADA2 v1.16 pipeline [34]. The assembled ASVs were then assigned taxonomy at the genus and species level using a custom formatted reference database constructed using the taxa included in the mouse oral microbiome database (MOMD v5.1) available at http://www.momd.org. The generated ASV counts were normalized for sequencing depth using the median of ratios method in the DeSeq2 [35] package in R, followed by beta diversity and relative abundance analyses of the microbial population. Graphical analysis and plots were created using the R packages phyloseq and ggplot2 [36].

### Single-cell transcriptomics preparation, sequencing, and analysis

Wild-type *CD1 wild type* mice (n = 5 group) were used for cell isolation from maxillary molars for single-cell transcriptomics experiments. The mice used for single-cell experiments were 8 weeks-old at start of the experiment and 14 weeks-old at the end. Group 1 (Homeostasis with Water or Metformin): The mice were put on either water or Metformin when they were 8 weeks old. As these groups were not inoculated, the animals stayed on water or Metformin for a total of six weeks. Group 2 (Periodontal disease induction with Water or Metformin): The mice groups that were inoculated were either on water or Metformin for four weeks prior to inoculation and remained on the treatments two weeks after inoculation, having a total six weeks of treatment. At the end of the experiment, the mice were sacrificed by neck dislocation and the maxillae were carefully dissected and under stereomicroscope. Using scalpel and tweezers, the maxillary gingival neck was carefully excised [37]. The soft tissues on the buccal area of the gingival tissues were carefully handled during excision. A total of 10 excised gingivae per group were used for processing and sequencing. Following dissection, the mouse gingivae were cut in small pieces and transferred to a 15 ml falcon tube with PBS + 2% FBS with 2 mg/ml of Collagenase Type II and 1 mg/ml of DNAse Type I (Merck), and incubated for 20–30 min at 37 °C shaking (120 rpm) with homogenization of the suspension every 3–4 min. After incubation, the cell suspensions were toped up to 12 ml using using PBS + 2% FBS and filtered in using a 70 µm cell strainer. These were subsequently centrifugated in 4 °C precooled centrifuge for 5 min at 300 × *g*. The pellet was then re-suspended in 100 μL of PBS + Ultrapure^TM^BSA (0.04%) (Thermo-Fischer Scientific) and sent to the BRC Genomic Centre where the library preparation, sequencing, and analysis were performed.

Single-cell suspensions were manually counted using a haemocytometer and concentration adjusted to a minimum of 300cells/μL. Cells were loaded according to standard protocol of the Chromium single-cell 3ʹ kit to capture around 5000 cells per chip position. Briefly, a single-cell suspension in PBS 0.04% BSA was mixed with RT-PCR master mix and loaded together with Single Cell 3ʹ Gel Beads and Partitioning Oil into a Single Cell 3ʹ Chip (10 × Genomics) according to the manufacturer’s instructions. RNA transcripts from single cells were uniquely barcoded and reverse transcribed. Samples were run on individual lanes of the Illumina HiSeq 2500.

Fastqs for each sample were generated and were demultiplexed using bcl2fastq software. The quality of the fastq files were checked using fastqc software. The analysis was carried out using Cellranger version 7.0.1, a proprietary software from 10 × genomics. Cellranger count module was run to analyse each sample fastq files using Mouse (mm10) reference (generated from GENCODE vM23/Ensembl 98) downloaded from 10 × website. A web summary file along with other intermediate files were generated for each sample. Cellranger aggregate module was used to aggregate all the 4 samples together using the h5 files from count output. A sequencing depth normalization is applied to all the samples during aggregate step to reduce any artifact from various sequencing depths of the samples by cellranger. The aggregated cloupe file was loaded into Loupe Browser version 6 for downstream analysis and visualizations.

The dimensionality reduction of the cells was carried out using PCA. A total of 9 clusters were generated using GRAPH based clustering method, which uses sparse nearest-neighbor graph and Louvain Modularity Optimization algorithms. UMAP and t-SNE were used to visualize the data in loupe browser.

A negative binomial test was carried out by cellranger to identify the genes which are expressed specifically to each cluster. The clusters were annotated with respect to the expressed markers. Reclustering was performed for the annotated Epithelial, Mesenchymal, and Immune cell clusters and quality control performed by filtering the cells with Mitochondrial counts > 10% and cells with unique features per barcode > 3000.

Local distinguishing differential expression test were carried out between the groups using loupe browser. Top 100 differentially expressed genes with adjusted p-value < 0.05 by Wilcoxon Rank Sum test featuring in more than 1 count per cell across the cluster set were used for further analysis.

Gene ontology (GO) analysis was performed on the selected top 100 differentially expressed genes using ShinyGO (v0.77), a R-shiny webserver. Top enriched GO Biological Process, Cellular Components and Molecular Function terms were generated from the genelist against Ensemble mouse gene sets. GO terms with an FDR < 0.05 were reported along with the dot plots for top 10 non redundant GO terms.

### Clinical trial

This study was reported following the Consolidated Standards of Reporting Trials (CONSORT) 2010 guidelines (Additional file 8: Fig. S1). The study was a single-centre, parallel group, double blinded pilot randomized controlled clinical trial with 6 and 12 weeks follow up. The study protocol was approved by the ethical committee of the University São Leopoldo—MANDIC (Brazil) (Approval code: 4.719.922) through the “Plataforma Brasil” (40340320.1.0000.537 4) and registered in the public platform REBEC (UTN code: U1111-1276-1942).

### Study design

This study was set to investigate the effect of Metformin on acute systemic inflammation during Periodontal Treatment in healthy non-Diabetic patients. Twenty patients in good health and diagnosed with Generalized Periodontitis stage 3 or 4 Grade B or C, requiring for non-surgical periodontal treatment were selected to participate in the trial. The volunteers were randomized into two groups: control group (Placebo, n = 10), test group (850 mg oral Metformin, n = 10). Participants were instructed to start the administration of medication (1 oral tablet) once a day, orally, in the morning at breakfast, 3 days before periodontal treatment and after blood and gingival crevicular fluid (GCF) collection for baseline laboratory tests. Full mouth non-surgical treatment (FMNST) was provided to the participants on the third day of medication. During treatment, the granulation tissue of the deepest periodontal pocket was collected. After treatment, the medication was prescribed once a day, orally, in the morning at breakfast, for 7 days, similarly to the prescription course of antibiotic therapy. In total participants had 10 days of either Placebo or 850 mg Metformin (Additional file 9: Fig. S2).

### Participants

All individuals attending the Unit of Periodontology at the University São Leopoldo—MANDIC (Brazil) were screened for eligibility. Subjects were eligible to participate if they met the following inclusion criteria: (i) age 18 years and above; (ii) Fit and well, without current or previous diagnosis of diabetes mellitus (Glycosylated haemoglobin > 6.5%), confirmed by blood Basal insulin and Fasting glucose levels; (iii) Patients requiring periodontal treatment diagnosed to stage 3 or 4 generalized periodontitis, grade B or C; (iv) Body Mass Index (BMI) within range of normal to overweight (BMI ≥ 18.5 ≤ 29.9 kg/m2). All participants were provided with the subject information sheet and gave written informed consent, which was followed by collection of medical and dental histories, Blood, and Gingival Crevicular Fluid sample collection and comprehensive oral examination.

We excluded participants undergoing cancer treatment; taking immunosuppression medication; alcoholics; smokers; with renal failure; liver changes; congestive heart failure; history of acute myocardial infarction less than 6 months ago; respiratory changes (pneumonia, pulmonary embolism, asthma, chronic obstructive pulmonary disease); allergy to any component of the medication formula; when using any medication that may interfere with the protocol of treatment, for example anti-inflammatories or antibiotics; and individuals who do not agree to sign the informed consent.

### Randomization

Participants were randomly allocated in a 1:1 ratio to receive either 850 mg Placebo or 850 mg Metformin as an adjunct to Full Mouth non-surgical therapy (FMNST) by using a computer-generated random sequence. Allocation to treatment was concealed in consecutively numbered opaque envelopes and revealed to the therapist and patients at the end of the last visit of the last patient of the trial.

### Periodontal treatment

All patients received Step 1 and Step 2 of treatment according to EFP guidelines [38]. Thus Step 1 was implemented with oral Hygiene Instructions (OHI) and motivation sessions. Patients were instructed to use a rotating oscillating toothbrush (Oral B®) and interproximal brushes; OHIs were reinforced during each treatment session and follow up visits. Supra and subgingival mechanical instrumentation (Step 2) of the root surface was performed by a single periodontist, using both hand and ultrasonic instrumentation with fine tips; local anaesthesia was performed wherever needed. The FMNST was performed within a single session. All patients were reviewed at Day1, Day 3, Day 7 after FMNST was performed. Periodontal reassessment was performed 6 and 12 weeks after FMNST.

### Bulk RNA sequencing

Granulation tissues were collected on the day of treatment, placed in 100 μl of RNAlater, flash frozen, and stored in − 80 °C. Total RNA was obtained using the “Quick- RNA MicroPrep” kit (Zymo Research) according to manufacturer's instructions. Following this, total RNA was sent to the Oxford Genomics Centre (Oxford, United Kingdom) for quality control, library preparation and sequencing using Illumina NovaSeq6000 (150 bp, pair-ended) platform.

The fastq reads where aligned to the human genome (GRCh38.p14/hg38) using an RNAseq specific aligner. To quantify gene expression levels, count tables were generated using the human hg38 transcriptome as reference. The count tables of each sample group were then compared and subjected to expression testing to identify differentially expressed genes between the two conditions. Raw reads were mapped to GRCh38.p14/hg38 using Hisat2, and EdgeR together with the Cufflinks pipeline were used to identify genes with differential gene expression. A gene was classified as being differentially expressed if it had a q < 0.05.

Gene Ontology (GO) functional annotation and KEGG pathway enrichment analysis for the up and down-regulated expressed genes in the granulation tissues was performed using Enrichr database. Three categories, including biological process (BP), molecular function (MF), and KEGG pathway were included in GO annotation. The top 10-enriched GO items and KEGG pathways were displayed on the webpage and downloaded as images.

### Blood sample collection

Blood samples were collected from a venepuncture in the antecubital fossa before 8am after an overnight fast for all participants. Blood samples were collected at baseline (3 days prior to FMNST) before medication was disposed, 1 day after FMNST, and 7 days after FMNST. Blood samples were processed and analysed by the ISO-certified laboratory at the University São Leopoldo—MANDIC (Campinas, Brazil) by an operator blinded to group allocation. Full blood count, high-sensitivity C-reactive protein (CRP), interleukin-6 (IL-6), basal insulin, glucose, erythrocyte sedimentation rate (ESR), Fibrinogen, and Tumour necrosis factor-α (TNF- α), were measured following standard laboratory procedures.

### Luminex

Gingival Crevicular Fluid (GCF) was collected using PerioPaper (Oraflow) from six sites with pocket probing depth ≥ 4 mm (3 maxillary sites and 3 mandibular sites), at baseline, 1 day, 3 days, and 7 days after treatment. The GCF strips were eluted and analysed by performing a multiple bead immunoassay technique (Luminex, Biotechne; R&D Systems). Eight GCF markers were assessed in total: IL-6, Insulin, VEGF, Angiogenin, C-Reactive Protein/CRP, IL-10, Leptin/OB, and Vitamin D.

### Diet diary

Participants were asked to record their daily food consumption (what they consumed and what time they consumed it), for the 10 days they were prescribed the medication. The participants were a semi-structured template to record their food consumption that allocated foods in three main meals (Breakfast, Lunch, Dinner). Participants were not asked to annotate quantity. Each element (food) of their daily diet annotated in the diary was counted as “1 serving” and the food group allocation was done according to the UK Eatwell Guide’ (EWG) (a policy tool to define government recommendations on eating healthily and achieving a balanced diet).

### Periodontal clinical parameters

At baseline and reassessment examination, all participants received a full periodontal evaluation by a single masked calibrated examiner. One examiner was trained for repeatability for Clinical Attachment Level (CAL) measurement. Periodontal parameters such as PD and recession (REC) were assessed full-mouth and rounded to the nearest millimetre (UNC15 mm periodontal probe); CAL values were obtained by the sum of PD and REC. Plaque and Bleeding on Probing (BoP) were recorded dichotomously six sites per tooth in order to calculate the Full Mouth Plaque Score (FMPS) and the Full Mouth Bleeding Score (FMBS), respectively.

### Statistical analysis

All statistical analyses were conducted using Graphpad Prism 9.5.0 at an alpha level of 0.05. Prior to conducting statistical analyses, normality assumptions were assessed using the Shapiro–Wilk test. The Shapiro–Wilk test was conducted on the data collected from the two arms of the trial. A p-value of less than 0.05 was considered to indicate a departure from normality. If the normality assumption was not met, non-parametric tests were used for subsequent statistical analyses.

To test for differences in normal datasets, Unpaired T-test was conducted for intergroup analysis and repeated measures one-way ANOVA, with post-hoc Tukey multiple comparison test was used to test differences across multiple timepoints within each arm of the trial.

To test for differences of non-normal datasets, a Mann–Whitney test was conducted intergroup analysis and a Friedman test, with post-hoc Dunn’s multiple comparison test was used to investigate significant difference in the medians of the variable across the different time points of each arm of the trial.

## Results

### Metformin disrupts periodontal disease establishment and progression in vivo

**Fig. 1:**
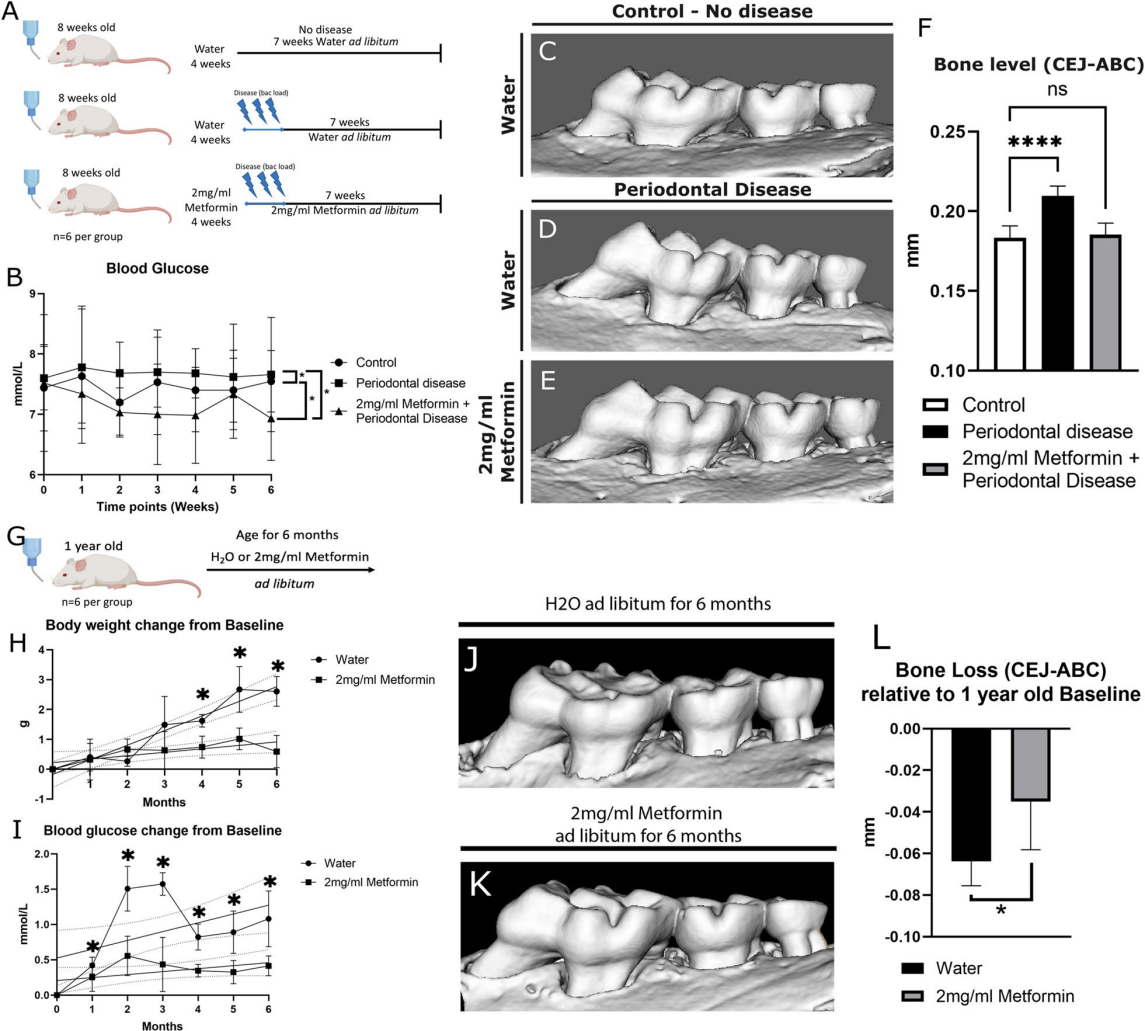
Preventive Metformin preclinical models. **A** Periodontitis induction experimental plan (Created with BioRender.com). **B** Longitudinal blood glucose levels prior (0) and 6 weeks post bacterial inoculation. **C–E** μCT 3D reconstructions of groups displayed in A. **F** Vertical bone loss analysis showing bone levels. **G** Longitudinal age-related bone loss experimental plan (Created with BioRender.com). **H** Body weight gain over 6 months relative to baseline (dotted lines indicates 99% confidence bands). **I** Blood glucose fluctuation over 6 months relative to baseline (dotted lines indicates 99% confidence bands). **J**, **K** μCT 3D reconstructions of ageing mice. **L** Vertical bone loss analysis showing bone levels relative to baseline measurements. Statistics **B** RM ANOVA (Between treatments) F (1.384, 8.307) = 19.33, *p* = 0.0013; Tukey Multiple comparison tests: Control vs Periodontal Disease: *p* = 0.0062; Control vs Metformin Prevention: *p* = 0.008; Periodontal Disease vs Metformin Prevention: *p* = 0.004. (**F**) T test: Control vs Periodontal Disease: *p* = 0.0096. **H** Simple Linear Regression: Slopes: F = 41.3.DFn = 1,DFd = 80. P < 0.0001; T test: Month 4 Water vs Metformin: *p* = 0.0061; Month 5 Water vs Metformin: *p* = 0.0078; Month 6 Water vs Metformin: *p* = 0.0002. **I** Simple Linear Regression: Slopes: F = 3.457.DFn = 1,DFd = 80.* p* = 0.0667; Intercepts: F = 43.63.DFn = 1,DFd = 81. P < 0.0001; T test: Month 1 Water vs Metformin: *p* = 0.0154; Month 2 Water vs Metformin: *p* = 0.0087; Month 3 Water vs Metformin: *p* = 0.0028; T test: Month 4 Water vs Metformin: *p* = 0.002; Month 5 Water vs Metformin: *p* = 0.0177; Month 6 Water vs Metformin: *p* = 0.0218. **L** T test: Water vs Metformin: *p* = 0.0337

We set out to investigate the effect of GM modulation on PD development when induced using *Porphyromonas gingivalis* (*P.gingivalis*) oral gavage in mice. We aimed to understand the effect of using Metformin preventively to disease establishment on oral parameters and systemic associated risk factors (Fig. 1A).

After *P. gingivalis* inoculation, blood glucose was monitored for 6 weeks and animals managed with preventive Metformin maintained significantly lower blood glucose levels compared to controls (− 0.29 mmol/L; *p* = 0.0062) and the animals that underwent periodontal disease induction (− 0.51 mmol/L; *p* = 0.004). Furthermore, animals that had disease induced but no treatment had a significantly higher blood glucose levels (+ 0.22 mmol/L; *p* = 0.008) (Fig. 1B). Intraorally, μCT 3D reconstructions of the molar area showed that *P.gingivalis* inoculation induced significant bone loss in animals without treatment (− 0.026 mm;* p* < 0.0001), whereas preventive management with Metformin halted *P.gingivalis*-associated bone loss, with bone levels remaining similar to that of the control group (− 0.001 mm;* p* = 0.8433) (Fig. 1C–F).

Since Metformin demonstrated a preventive potential in an induced PD model, we further investigated the effect of using Metformin on naturally occurring age-associated periodontal bone loss (Fig. 1G). Linear regression showed that long-term use of Metformin (6 months) significantly controlled age-related weight gain (*p* < 0.0001), with animals on drinking water being four times heavier than those on Metformin at the end of the experiment (Fig. 1H). Moreover, long term use of Metformin maintained glucose levels stable at a lower level when compared to non-treated animals. Linear regression showed that the intercepts of the glucose levels slopes were significantly different (*p* < 0.0001) (Fig. 1I). Intraorally, μCT analysis showed that long-term use of Metformin significantly prevented 54% of the age-related bone loss (0.035 mm) when compared to non-treated animals (0.064 mm) (*p* = 0.0337) (Fig. 1J–L).

### Metformin affects the host-microbiome axis

**Fig. 2:**
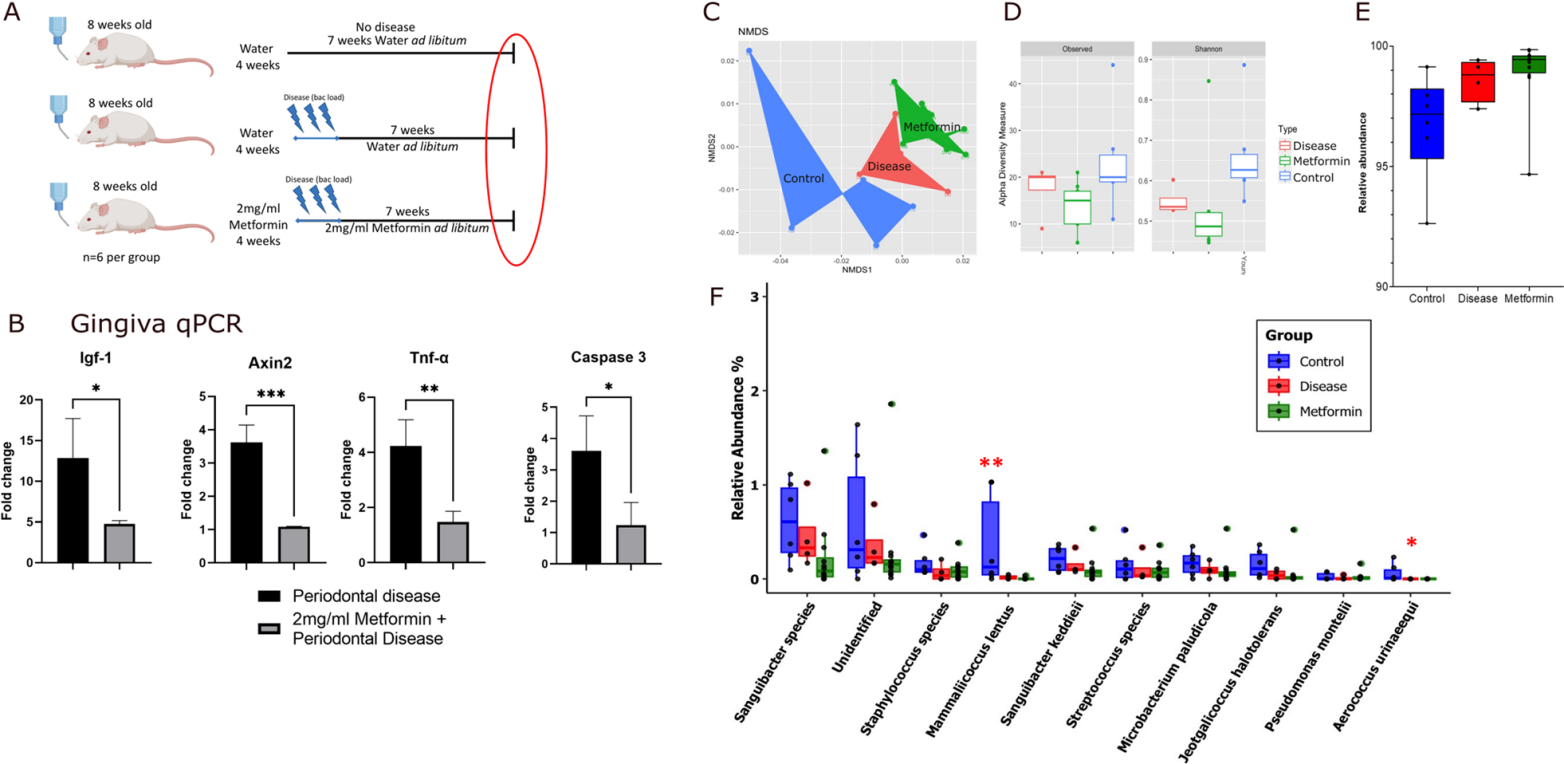
Gingival inflammation and microbiome composition. **A** Red circle indicates the time point of the analysis (Created with BioRender.com). **B** qPCR of gingival tissues from mice treated and not treated with Metformin relative to control animals (without disease induction). **C–F** 16 s rRNA gene amplicon sequencing of the oral microbiome of the three groups presented in A (n = 6). **C** Beta diversity, **D** Alpha diversity, **E** Adonis test with *S. danieliae*, **F** Adonis test of the ten most abundant taxa following exclusion of *S. danieliae*

To test the effects on the gingival tissues and oral microbiome, gingival tissues were dissected, and oral swabs were collected from the animals that underwent periodontal disease induction at the end of the experiment. These samples were used for qPCR analysis and 16 s rRNA gene amplicon sequencing of the oral microbiota (Fig. 2A).

Metformin-treated gingiva showed significantly lower gene expression levels of inflammatory markers related to periodontal disease progression [39] Tnf-α (− 2.7 fold; *p* = 0.005) and Caspase 3 (− 2.3 fold; *p* = 0.011), as well as lower expression of reparative markers important to reconstruct the periodontium [40] Igf-1 (− eightfold; *p* = 0.036) and Axin2 (− 2.5 fold; *p* = 0.004), suggesting a more normal (homeostatic) gingival tissue (Fig. 2B).

To confirm that our *P. gingivalis* gavage model generated dysbiosis in the oral environment and assess whether Metformin affected the oral microbiome, 16 s rRNA gene amplicon sequencing of the oral microbiota targeting the V1-V2 variable regions was employed. Beta-diversity analyses of the oral microbial populations of the sampled mice showed distinct separation in the clustering of all three groups (Fig. 2C). Alpha-diversity analysis showed significant differences between microbial populations in the animals that were exposed to Metformin compared to diseased and controls (Pairwise Wilcoxon Test: Metformin Diseased vs Water Diseased *p* = 0.0264; Metformin Diseased vs Control *p* = 0.0061) (Fig. 2D). At the species level, the organism *Streptococcus danieliae* is known to be most abundant in the mouse oral cavity, with a relative abundance of greater than 90% in most conditions [24]. Non-parametric ANOVA/Kruskal–Wallis test showed a significant variation in the abundance of this organism between the three groups (*p* = 0.0087) (Fig. 2E). Since the high levels of this organism can mask other variations in the microbial population, the ten most abundant taxa following exclusion of *S. danieliae* were also analyzed. Based on Adonis testing of the entire population, there was a significant difference between the three groups, suggesting that the treatments have an effect on the compositional difference of the oral microbiome. ANOVA/Kruskal–Wallis testing showed a significant variation between the groups for the species *Mammaliicoccus lentus* (*p* = 0.013) and *Aerococcus urinaeequi* (*p* = 0.015) (Fig. 2F)*.*

### Metformin enhances gingival metabolic response in early disease development at single cell level

To provide an in-depth analysis of the effect of Metformin preventive effect on gingival cell dynamics when transitioning from health to disease, we transcriptionally profiled single cells derived from mice that underwent Metformin treatment in health and PD induction. We obtained freshly dissected mouse gingival tissue and isolated live cells to be sequenced on the 10 × Genomics Chromium platform for single-cell RNA-seq (scRNA-seq) (Additional file 10: Fig. S3A). A total of 8,295 cells were captured across four treatment groups, allowing us to perform an in-depth analysis of single-cell transcriptomics. This analysis revealed nine distinct transcriptomic signatures correspondent to the cell types visualised using UMAP (Additional file 10: Fig. S3B–E, Additional file 1, 2 and 3). Our murine gingival UMAP is comparable to previously published human gingival UMAP distribution, with a fewer cell subtypes captured due to the depth of analysis [41].

**Fig. 3:**
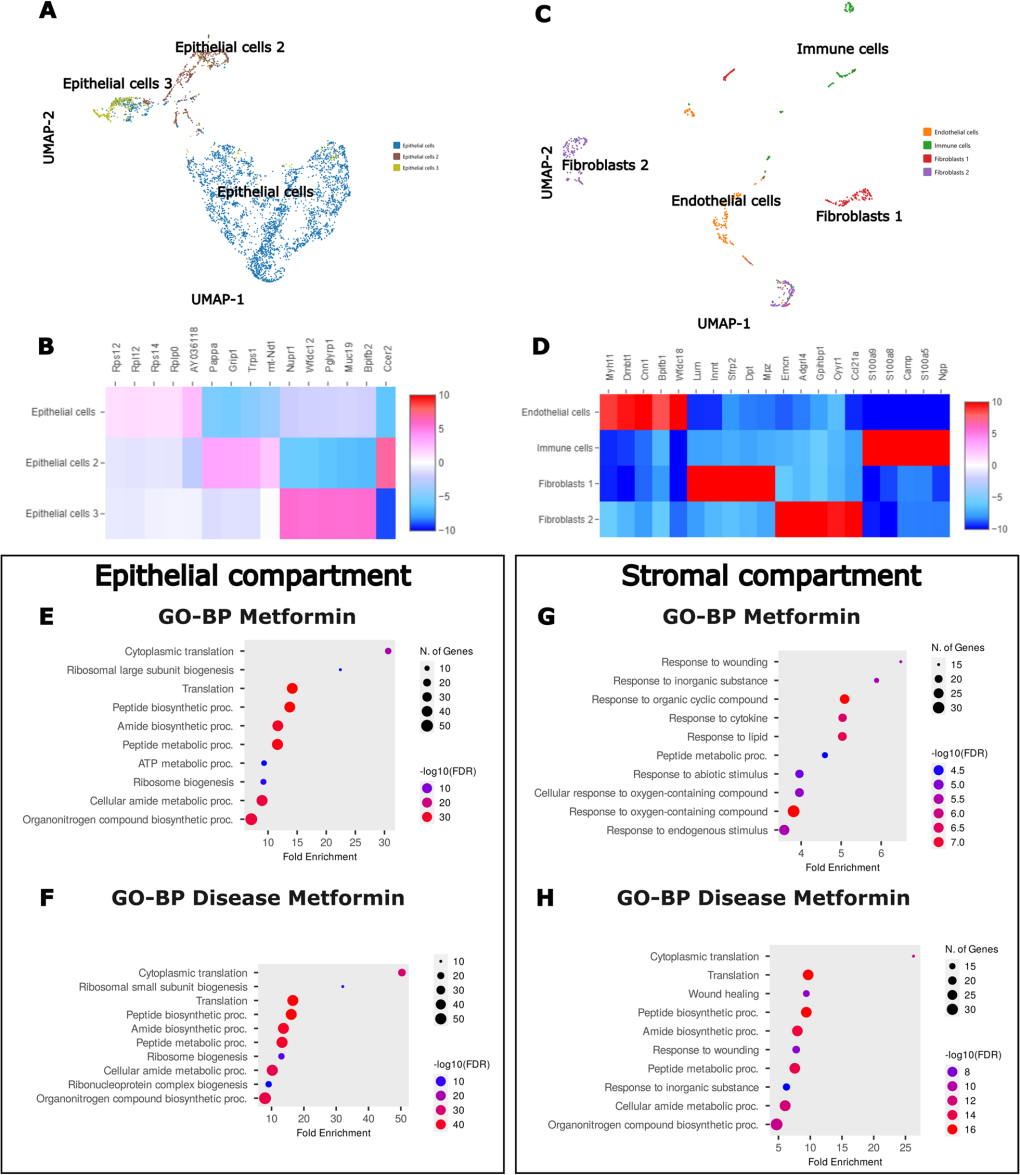
scRNAseq analysis of gingival epithelial and stromal compartments during Metformin treatment. **A**, **B** UMAP plot of gingival epithelial cells with heatmap showing subset-specific markers (4280 cells). **C**, **D** UMAP plot of gingival stromal cells with heatmap showing subset-specific markers (783 cells). **E–H** Dot plots depicting GO enrichment terms for BP of epithelial and stromal cell compartments of upregulated genes when treated with Metformin with and without disease induction

To better visualise the tissue-specific effects of Metformin in health and disease, re-clustering analysis of epithelial and stromal cells was performed to identify the effects of Metformin on sub-clusters transcriptional signature (Fig. 3A–D). Differential analysis of the clusters in different conditions was performed (without considering genes with low average count) and the first 100 upregulated genes were enriched by gene ontology (GO). GO terms associated with metabolic process, energy metabolism, upregulation of translation, and wound healing were detected in epithelium and stromal cell clusters when mice were treated with Metformin. This was found in both homeostasis (No disease + Metformin) and during disease response (Metformin + disease) (Fig. 3E–H). Interestingly, Metformin treatment upregulated metabolism related GO signatures which were conversely downregulated in epithelial and stromal cells when Metformin was not administered prior to disease induction (Additional file 11: Fig. S4A).

Further analysis of the dataset according to the GO related genes revealed significant differences in gene expression in the epithelium and stromal compartments. We first analysed the epithelial and stromal compartments of the animals that did not undergo disease induction. In the epithelium, when compared to the water control, Metformin significantly upregulated Calml3 (a gene important for inhibition of cell apoptosis during oxidative stress [42]), Pde4d (a regulator of cellular metabolism and energy homeostasis through cAMP [43]), Krtdap (important for keratinocyte differentiation and maintenance of stratified epithelia [44]), and Rpl10 (a mitochondrial modulator of reactive oxygen species (ROS) [45]) (Additional file 12: Fig. S5A, Additional file 16: Table S1, Additional file 4). In the stromal compartment, when compared to the water control, Metformin significantly upregulated Angptl1 (a glycoprotein shown to inhibit osteoclast formation and osteoblastic differentiation [46]), Ifitm3 (involved in immune response or antiviral activity [47]), Ncp2 (a gene encoding a protein responsible for cholesterol binding, shown to be important in periodontal tissues homeostasis [48]), and Serpinh1 (a controller of endoplasmic reticulum stress, responsible for maintaining cell homeostasis under high glucose stress [49]) (Additional file 13: Fig. S6A, Additional file 16: Table S1, Additional file 5).

Following that, we investigated the effect of preventive Metformin on the epithelial, stromal and immune cell compartments of animals that underwent PD induction. We first investigated the differences between diseased epithelium and stromal tissues against healthy control epithelial and stromal tissues. Our results showed that in the diseased animals, those cell compartments suffered metabolic changes with significant downregulation of genes associated with the control of ROS damage and glycolysis (Prdx6, Gstm1, Cbr2, Ldhb), mitochondrial respiration and energy (mt-Nd3, mt-Nd2), and cell structural genes (Krt4, Tuba1a) (Additional file 11: Fig. S4B, Additional file 6).

Further, when comparing the group that underwent preventive Metformin administration versus the group on water prior to disease induction, our results showed that in the Metformin group, the epithelial cells upregulated Krt76 and Krt6a (genes important for epithelial immune reaction and differentiation [50]), Gas5 (a gene important for glucocorticoid receptor control which modulates insulin resistance [51]), and Fxyd3 (a regulator of Na + /K + ATPases, maintaining osmotic equilibrium and membrane potential in epithelial cells [52]) (Additional file 12: Fig. S5B, Additional file 16: Table S1, Additional file 4). In the stromal compartment, the preventive Metformin group significantly upregulated Atp2a2 (a gene encoding a key regulator of intracellular calcium ions shown to inhibit aberrant cell growth [53]), Myh11 (important in the provision of information for endothelial contraction and related to suppression of diabetes-accelerated atherosclerosis [54]), the insulin signalling pathways Igf1r and Sorbs1 (important for maintenance of proliferative capacity of fibroblasts [55] and insulin-stimulated glucose uptake [56], respectively) (Additional file 13: Fig. S6B, Additional file 16: Table S1, Additional file 5). Finally, sub analysis of the inflammatory cells in the stromal compartment showed that preventive Metformin enhances genes related to bacterial defence, apoptosis regulation, metabolism and ATP synthesis, corroborating with aforementioned gene expression analysis of the gingiva (Additional file 14: Fig. S7, Additional file 16: Table S1, Additional file 7).

Overall, we demonstrated in silico that Metformin was capable of enhancing the diseased epithelial and stromal cell compartment metabolism towards homeostasis. Moreover, Metformin primed the gingiva with a protective capacity to oxidative damage and via increasing cell energy. Collectively, these observations suggest that Metformin treatment acts as a preventive modulatory drug against PD establishment at the molecular level of the disease pathogenesis.

### Systemic Metformin modulates acute systemic inflammation in non-diabetic periodontal patients

Systemic Metformin is widely used in the management of diabetes (and for non-diabetic treatments, such as Polycystic Ovarian Syndrome), however, it has never been used in periodontitis treatment management. Therefore, in order to investigate whether Metformin affects systemic-oral inflammation, an associated risk factor of the pathogenesis of PD [57], a pilot randomized clinical trial was developed as a first step attempt to repurpose Metformin for use in PD management in non-diabetic periodontal patients. For the human clinical study, 521 individuals were screened for eligibility, and 20 individuals were enrolled in the study. These patients, were diagnosed with Generalised Periodontitis Stage III Grade B (n = 4/group) and Generalised Periodontitis Stage IV Grade B (n = 6/group), according to the European Federation of Periodontology. No participant was lost to follow-up appointments, and all samples were included in the final analysis. Our participants were healthy, non-smokers, non-diabetic, and with BMI ranging from normal to overweight; no significant differences were seen between groups (Additional file 16: Table S2).

Since the recruited patients were non-diabetics, careful tracking of any adverse effects from the off-label use of Metformin for periodontal treatment was done. During the medication window, 4 participants reported adverse effects, two in each group (Additional file 16: Table S3). However, the adverse effects reported were not sustained during the whole medication window, but only lasted less than 48 h.

**Fig. 4:**
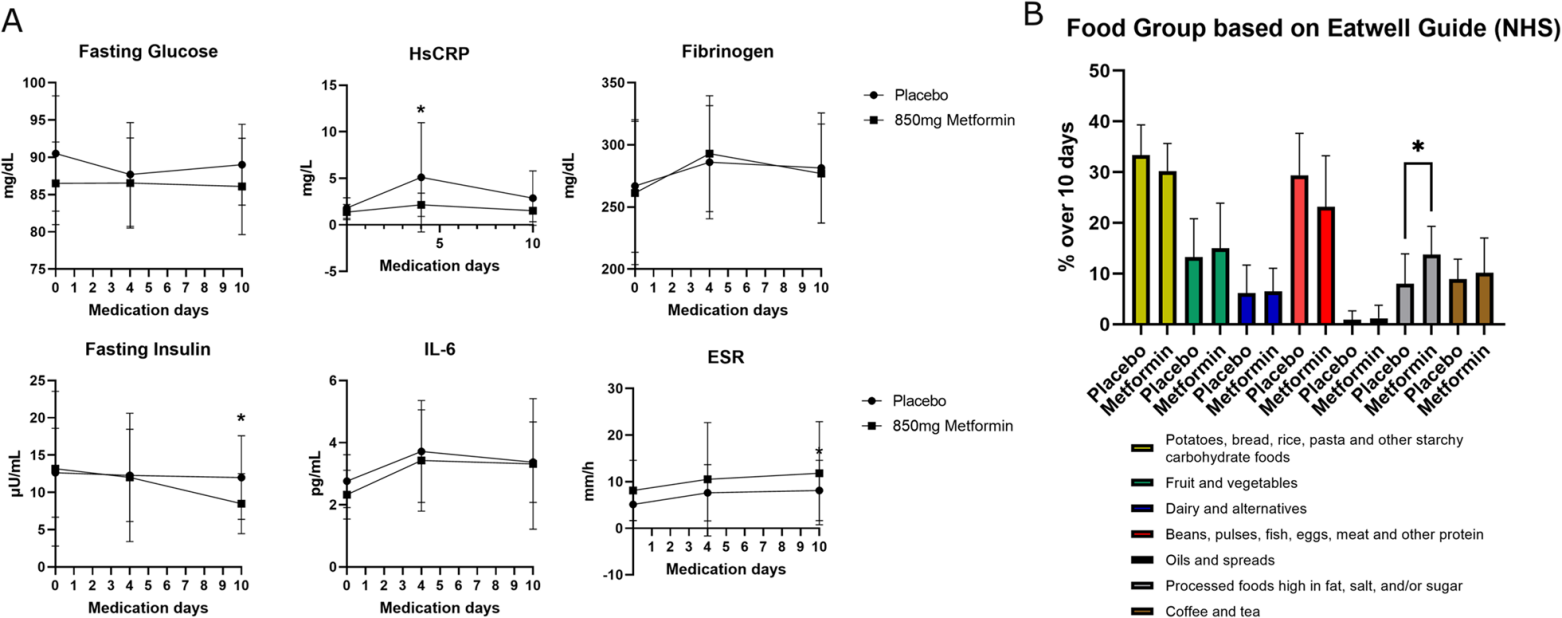
Acute systemic inflammatory reaction and diet during Metformin administration. **A** Blood systemic metabolic and inflammatory markers from baseline (day 0) until 1 week after periodontal treatment (day 10). Day 4 reflects the 1 day after periodontal treatment. **B** Diet analysis during the medication prescription. Statistics for blood systemic markers and diet diary are in Tables S3 and S4, respectively. Abbreviations: ESR, erythrocyte sedimentation rate; NHS, National Health Service

Periodontal treatment increases systemic inflammatory response in the first week post treatment [58], therefore, we evaluated the effect of Metformin on modulating systemic inflammation and metabolism during this acute inflammatory phase post treatment. The levels of systemic inflammatory and metabolic markers at baseline and during the medication window are shown in Fig. 4A and Additional file 16: Table S4. During the course of medication, Metformin-treated patients maintained a stable fasting glucose level from baseline. Moreover, the Metformin group showed significantly lower fasting insulin levels 7 days after treatment when compared to baseline (10th day of medication: − 4.68 μU/ml (Mean); *p* = 0.0303).

The primary outcome of the trial was to determine any differences in acute phase elevations in levels of circulating C-reactive protein, assessed by high-sensitivity C-Reactive Protein assay (HsCRP) after treatment, and although no intergroup differences were seen, HsCRP levels were significantly elevated only in Placebo-treated patients in the intragroup analysis (+ 3.29 mg/dL; *p* = 0.0313). Additionally, ESR was significantly elevated only in Placebo-treated patients (*p* = 0.0460), whereas circulating Basophils and Lymphocytes levels were significantly elevated only in Metformin-treated patients at day 7 after treatment when compared to baseline (Additional file 16: Table S4).

Since Metformin is a glucose metabolism modulatory drug, food consumption was investigated as a potential confounder in the analysis of systemic blood markers. Intergroup analysis of the diet diary collected from the participants showed that in general the participants had a similar diet and consumed a similar number of servings over the 10 days of medication (Additional file 16: Table S5). However, the Metformin treated group reported having consumed significantly more servings of processed foods high in fat, salt, and/or sugar than the placebo group (+ 5.74%; *p* = 0.0375) (Fig. 4B).

### Systemic Metformin effects on the periodontium during initial phase of healing

Systemic Metformin has never been used for periodontal treatment management in non-diabetic patients. Therefore, we investigated whether systemic administration of Metformin affected the periodontium and modulated periodontal responses during acute healing phase (initial week post treatment).

**Fig. 5:**
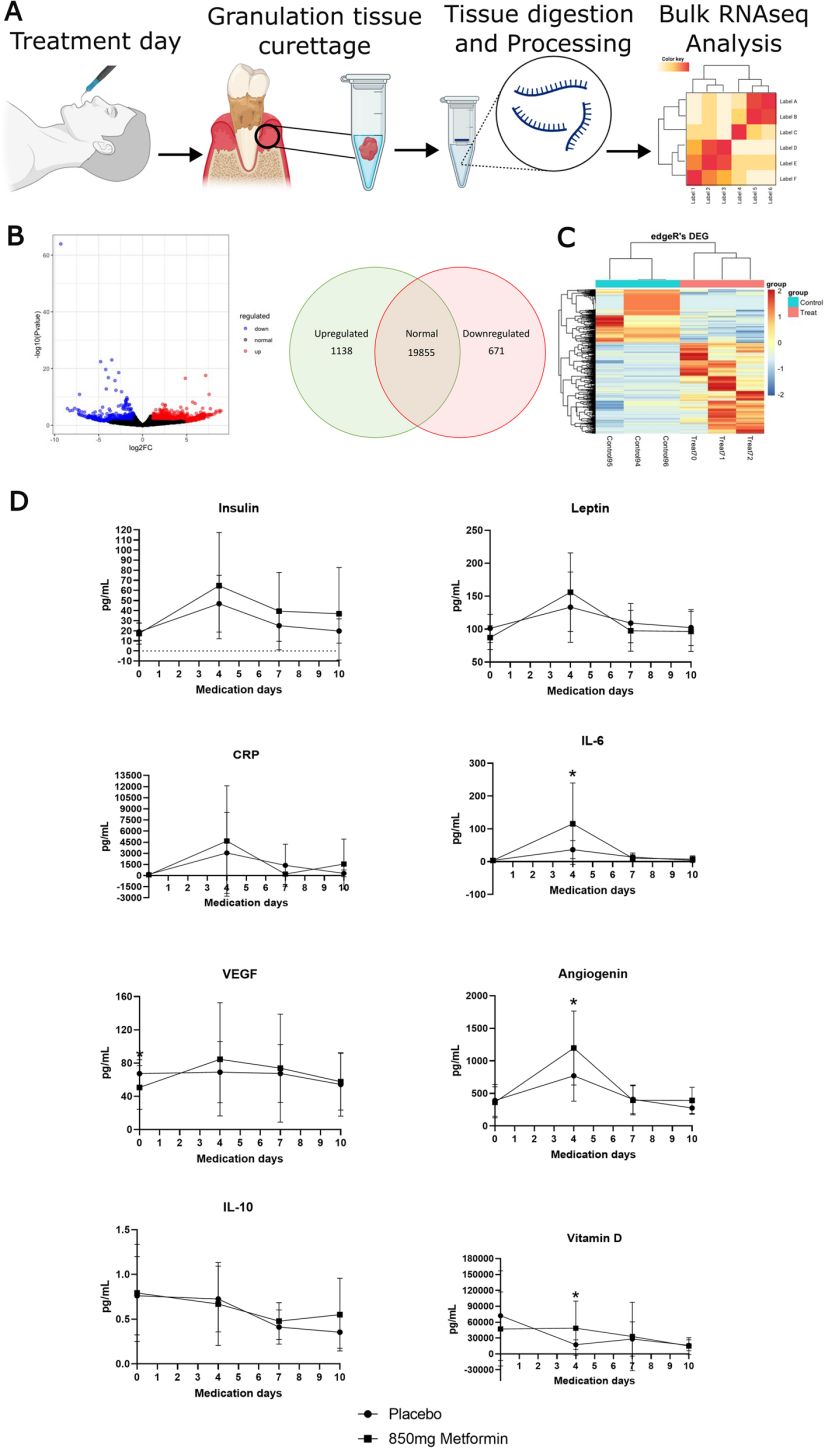
The effect of Metformin on the periodontium before and after treatment. **A** Schematic representation of the process from tissue collection via curettage for sequencing (n = 3 group) (Created with BioRender.com). **B** Differential expression testing between samples. Volcano plot showing the results of differential expression testing and Venn diagram showing that Metformin upregulated 1138 genes and downregulated 671 genes in the granulation tissue by the day of treatment. **C** Heatmap displaying the different transcriptomic expression between treated and non-treated patients. **D** Gingival crevicular fluid analysis by Luminex Multiplex Assay at baseline (day 0) and during the initial healing phase (Statistics for Luminex in Additional file 16: Table S6) (Day 4 of medication = 1 day after treatment; Day 7 of medication = 3 days after treatment; Day 10 of medication = 7 day after treatment)

On the day of periodontal treatment, participants undergoing non-surgical periodontal therapy had their chronic inflammatory granulation tissues collected for bulk RNAseq analysis (Fig. 5A). Differential expression testing was performed to identify genes whose expression level was statistically different between the Placebo- and Metformin-treated participants. The analysis revealed a distinct expression signature between treatments with 1,138 significantly upregulated genes and 671 significantly downregulated genes in Metformin-treated participants (Fig. 5B, C). GO Enrichment analysis on the upregulated genes in Metformin-treated participants, revealed that Metformin-treated tissues did not carry the same metabolic transcriptomic signature as the placebo granulation tissue, with the top enriched pathway in the Metformin group being related to “drug metabolism” (Additional file 15: Fig. S8A, B; Additional file 16: Table S6). Surprisingly, the top Metformin downregulated GO signatures were associated with viral defence, a microbial factor previously described as a key player in the chronicity of periodontal disease (Additional file 16: Table S5) [59].

Following periodontal treatment, the gingival inflammatory exudate derived from the periodontal tissues was analysed to understand the impact of systemic Metformin treatment on periodontal inflammatory response (Fig. 5D; Additional file 16: Table S7). The glucose metabolism hormones Insulin and Leptin elevated at day one after treatment, however, only Metformin-treated patients had significant changes in Leptin levels in the gingival crevicular fluid during the healing phase (*p* = 0.0074; 1D vs Baseline *p* = 0.0235). Increase of pro-inflammatory protein CRP and IL-6 are expected 24 h after FMNST [60]*.* Our results showed that such markers were more elevated in Metformin treated patients than the Placebo group. Only IL-6 showed significant elevation at day one after treatment in the Metformin-treated group (+ 78.98 pg/ml; *p* = 0.0326), however, in the following days both inflammatory markers normalised with the Placebo group. Since angiogenesis is an important step for wound healing, we investigated the levels of VEGF and Angiogenin. No significant changes were seen in VEGF levels during healing, however, Angiogenin levels one day after treatment were significantly higher when compared to Placebo (+ 428 pg/ml; *p* = 0.0328). Further, the anti-inflammatory cytokine IL-10 showed significant decrease only in the Placebo-treated patients (*p* = 0.0262). Finally, Vitamin D levels were significantly lower 1 day after treatment in Placebo-treated patients (-31,125 pg/ml; *p* = 0.0376) (Additional file 16: Table S7).

Whilst the effects of systemic admiration of Metformin can be appreciated in the periodontium, it cannot be concluded that these effects are due to the bioavailability of the drug on the local tissues, therefore further research is needed to evaluate the uptake of Metformin in the periodontal tissue. Nevertheless, systemic Metformin administration affected the periodontal tissues modulating immunity, metabolism, and pro- and anti-inflammatory markers during the initial phase of healing.

### Systemic Metformin improves periodontal treatment outcomes

Periodontal treatment produced significant clinical benefits for all periodontal parameters in both groups (Table 1). Overall, Metformin treatment caused a difference in probing pocket depth (PPD) reduction of 0.21 mm (median) and clinical attachment level (CAL) gain by 0.3 mm (median), 12 weeks after treatment. Further, at the initial reassessment (6 weeks post treatment), only Metformin-treated patients had a significant reduction in PPD when compared to baseline (Baseline vs 6 weeks after treatment: *p* = 0.0417), with an average reduction of 0.3 mm greater than the Placebo group, suggesting a potential acceleration in PPD reduction when participants took Metformin.

**Table 1:** Clinical periodontal outcomes

Variable	Groups	Initial	6 weeks Reassessment	12 weeks Reassessment	Intragroup Analysis †	Multiple comparison (1)	Multiple comparison (2)	Multiple comparison (3)
		Median (IQR)	Median (IQR)	Median (IQR)	p-value	p-value	p-value	p-value
PI (%)	Placebo	93.5 (6.5)	47.5 (5.25)	36.5 (17.25)	** < *0.0001***	***0.0076***	***0.0007***	> 0.99
	850 mg Metformin	98 (2)	47.5 (28.7)	55 (12.25)	** < *0.0001***	***0.0052***	***0.001***	> 0.99
	p-value between groups	0.3789	0.5657	0.1091				
BOP (%)	Placebo	37.5 (16)	5 (6)	1 (2.5)	** < *0.0001***	***0.0219***	***0.0002***	0.5391
	850 mg Metformin	43.5 (6.25)	4 (1)	2.5 (0.5)	** < *0.0001***	***0.0156***	***0.0003***	0.7907
	p-value between groups	0.9561	0.3383	0.8261				
PPD (mm)	Placebo	2.94 (0.36)	1.78 (0.27)	1.53 (0.34)	** < *0.0001***	0.9550	** < *0.0001***	0.0760
	850 mg Metformin	3.16 (0.26)	1.77 (0.47)	1.60 (0.32)	** < *0.0001***	***0.0417***	** < *0.0001***	0.2209
	p-value between groups	0.4696	0.955	0.7821				
PPD reduction (mm)	Placebo	N/A	1.13 (0.11)	1.43 (0.15)	–	–	–	0.0020*
	850 mg Metformin	N/A	1.43 (0.21)	1.64 (0.33)	–	–	–	0.0059*
	p-value between groups	–	0.1595	0.565				
CAL (mm)	Placebo	3.37 (0.54)	2.40 (0.54)	2.28 (0.38)	** < *0.0001***	***0.0417***	** < *0.0001***	0.2209
	850 mg Metformin	3.79 (0.16)	2.49 (0.10)	2.24 (0.16)	** < *0.0001***	0.0566	** < *0.0001***	0.1325
	p-value between groups	0.6162	0.7546	0.5659				
CAL gain (mm)	Placebo	N/A	1.02 (0.17)	1.36 (0.23)	–	–	–	0.0098*
	850 mg Metformin	N/A	1.36 (0.37)	1.66 (0.39)	–	–	–	0.0039*
	p-value between groups	–	0.1431	0.3527				
Pockets ≥ 5 mm %	Placebo	15.01 (7.80)	1.82 (1.33)	1.19 (1.59)	** < *0.0001***	***0.0156***	***0.0003***	0.7907
	850 mg Metformin	19.90 (9.27)	1.48 (1.71)	0.34 (0.53)	** < *0.0001***	***0.0219***	***0.0002***	0.5391
	p-value between groups	0.5419	0.9264	0.5072				

Bold values indicate statistical significance. Significant p-values (p < .05) are provided in italics

IQR, Interquartile range; PI, Plaque Index; BOP, Bleeding on probing; PPD, probing pocket depth; CAL, Clinical attachment level

Intergroup analysis: Mann–Whitney Test; Intragroup analysis: † Friedman Test, (1) Multiple compassion 6 weeks after treatment vs Baseline, (2) Multiple compassion 12 weeks after treatment vs Baseline, (3) Multiple compassion 12 weeks after treatment vs 6 weeks after treatment; *Wilcoxon Test

## Discussion

Glucose metabolism is widely known to directly impact PD progression [20, 21]. Nevertheless, little is known about how glucose metabolism impacts periodontal disease in non-diabetic individuals. Using non-diabetic preclinical models and periodontal patients, we discovered that Metformin (a pharmaceutical agent capable of modulating glucose metabolism) prevented periodontal disease establishment and progression, modulated metabolism and inflammation in the periodontal tissues, controlled acute systemic inflammation after periodontal treatment, and improved clinical periodontal parameters.

Periodontitis and gingivitis differ in the destruction of supporting periodontal tissues that is irreversible upon removal of microbial challenge. However, the trigger for progression from a stable chronic gingivitis to destructive periodontitis remains unclear [61]. Plaque accumulation is a physiological transient factor that affects the inflammatory response gingival tissues [62]. Additionally, glycemia is another physiological transient factor, which has been shown to affect the progression of diseases with overlapping inflammatory risk factors of periodontal disease [63]. Metformin is not typically used in dentistry, however, we showed in vivo that its systemic use maintained a stable glucose level and modulated the cellular energy metabolism as well as differentiation capacity of epithelial and stromal cells in the gingiva. This resulted in enhanced preventive capacity against periodontal disease establishment, even in the presence of dysbiosis.

Unfortunately, testing the same in vivo approach of bacteria-led gingival inflammation in patients (experimental gingivitis model) would not be ethical as systemic Metformin has never been used in the management of PD. Therefore, we developed the first trial to repurpose systemic Metformin as an adjunct to periodontal disease treatment, as a first step to investigate the potential use of Metformin in PD management. We showed that Metformin aids on control of systemic and local inflammatory response after treatment leading to a mild improvement in clinical periodontal parameters. Although we had a low n number (leaving the trial underpowered) and the medication was prescribed for only 10 days, the results were surprisingly satisfactory, giving signs of effectiveness of potential benefits of Metformin use as an adjunct to periodontal treatment. Another important consideration of our trial is the Metformin dose chosen. Our animals were administered a dose reported as the optimal oral dose for human diabetic treatment (1200 mg/60 kg/day) that resulted in enhancement of longevity [26–28], whilst our non-diabetic periodontal patients were prescribed a dose equivalent to 750 mg/60 kg/day, which is 1.6 times lower than the reported treatment for diabetes. Considering the transiency of plaque and glucose levels between treatment and reassessment and the low dose used, longer administration of Metformin could result in significantly improved periodontal clinical outcomes.

The optimal concentrations of systemic antibiotics used as adjuncts to PD management have been reported to improve PPD from a rage of 0.26–0.53 mm when taken after subgingival instrumentation [64]. Our clinical results demonstrate systemic Metformin, at the experimental course and dose used, improved 0.21 mm in PPD at 12 weeks after treatment. Such finding indicates a potential scope for dose and length of administration optimisation. Moreover, it is noteworthy that the plaque score of the patients under Metformin were higher than those on placebo, yet inflammation levels were low (signalled by Bleeding score), and % of pocket closure and clinical attachment gain were higher than placebo. In alignment to our animal model of dysbiosis, systemic Metformin enhanced periodontal parameters in the presence of high levels of bacteria. Additionally, in our clinical study, systemic Metformin acted on accelerating healing, seeing by significant decrease in PPD at 6 weeks. This corroborates with previous in vivo studies showing Metformin’s wound healing acceleration capacity [29, 65]. Therefore, our data indicates that Metformin’s action when given systemically for clinical periodontal treatment takes place via modulation of inflammation, but oxidative stress modulation may also be playing as role as described in Araujo et al. [29] and seen in our animal model*.* Finally, on a systemic level, Metformin administered as an adjunct to periodontal treatment stabilised fasting glucose and hsCRP, and decreased insulin levels, which may have also indirectly contributed for an improvement on the local effect seen in the biological and clinical parameters. Altogether, our data suggests that the use of Metformin for periodontal disease management could constitute a form of preventive medicine to oral and systemic health.

Long-term use of Metformin has been associated with decreased mortality and age-associated diseases [66]. Aging has been recently classified as a treatable disease by the WHO and targeting aging has been shown to be as economically viable as a horizon governmental policy [67]. Further, the prevention of periodontal disease establishment and progression was shown to be the best economical pathway as a governmental policy [68]. Our ageing in vivo model indicated that long-term use of Metformin has the potential to prevent periodontal and systemic comorbidities (diabetes and weight gain) that increase in incidence with age. Although further understanding of the effect of Metformin on adipose tissue percentage and body composition changes is needed, it is widely accepted that diabetes and weight gain play major role in inflammaging, a continuous increase in the general inflammatory status of the aging population [69, 70]. In return, this increase of inflammaging plays a role in increasing periodontal inflammation and disease severity [71]. Therefore, since a significant proportion of the population develops periodontal disease at a younger age (30 years old) in comparison to other age-related systemic non-communicable diseases, management of PD throughout life using glucose metabolism modulation strategies could result in indirect geriatric enhancement of overall health and wellbeing. Furthermore, it can move dentists away from overtly relying on antibiotics, a scenario that has recently been raising concerns about contributing to antimicrobial resistance [72].

In summary, systemic administration of Metformin in vivo provided an effective preventive effect on the pathogenesis of the periodontal disease establishment and progression by controlling systemic and periodontal metabolism, reaction to oxidative damage, and inflammation. Moreover, within the limitations of our pilot trial, Metformin appears to be a useful novel therapeutic adjunct option to periodontal treatment management, however, further testing with dose titration in larger trials are needed. The safety, readiness, and cost-effectiveness of this approach makes Metformin an ideal translational approach that could potentially be adopted on a global scale.

### Supplementary Information

**Additional file 1.** Significantly upregulated genes per cluster.

**Additional file 2.** Global distinguishing comparison between all samples using gingiva control as guide of expression- Upregulated genes.

**Additional file 3.** Global distinguishing comparison between all samples using gingiva control as guide of expression- Downregulated genes.

**Additional file 4.** Epithelial cluster comparison between Metformin treatment and no treatment- Significantly upregulated genes.

**Additional file 5.** Stromal cluster comparison between Metformin treatment and no treatment- Significantly upregulated genes.

**Additional file 6.** Downregulated genes in the comparison between healthy and diseased epithelial and stromal clusters.

**Additional file 7.** Immune cells cluster comparison between Metformin treatment and no treatment- Significantly upregulated genes.

**Additional file 8: Figure S1.** Flow chart of the study design according to CONSORT guidelines.

**Additional file 9: Figure S2.** Trial design flow diagram.

**Additional file 10: Figure S3.** Single cell clustering of mouse gingiva in health and disease induction.

**Additional file 11: Figure S4.** Downregulated transcriptome on epithelial and stromal compartment during disease initiation.

**Additional file 12: Figure S5.** UMAPs feature and violin plots mapping the significant upregulated expression code in the Epithelial compartment when Metformin is used during homeostasis and during early disease development.

**Additional file 13: Figure S6.** UMAPs feature and violin plots mapping the significant upregulated expression code in the Stromal compartment when Metformin is used during homeostasis and during early disease development.

**Additional file 14: Figure S7.** Sub analysis of immune cells clusters.

**Additional file 15: Figure S8.** Enrichr bar plots depicting GO enrichment terms and KEGG pathways for downregulated (Blue) and Upregulated (Red) genes.

**Additional file 16: Table S1.** Significantly expressed genes mentioned on text with P-values. **Table S2.** Inclusion criteria with periodontal and systemic parameters of both study groups. **Table S3.** Adverse effects reporting during the 10 days of medication of both study groups. **Table S4.** Systemic blood markers at baseline, 1 day after treatment (4 days of medication) and 7 days after treatment (10 days of medication). **Table S5.** Food diary evaluation during the 10 days of medication. **Table S6.** Up and Downregulated genes related to top enriched GO of bulk RNAseq. **Table S7.** GCF Luminex statistics.

## Data Availability

All data associated with this study are present in the paper or the Supplementary Materials. Requests for data should be addressed to the corresponding authors. Raw sequencing data obtained from animals and patients used in this study is deposited in the National Center for Biotechnology Information BioProject ascension: PRJNA950601.
