# Supplementary figures and images for: Repurposing Metformin for periodontal disease management as a form of oral-systemic preventive medicine
Source: J Transl Med. 2023 Oct 10;21:655. doi: 10.1186/s12967-023-04456-1 (PMC10563330; doi:10.1186/s12967-023-04456-1)

## CONSORT 2010 Flow Diagram

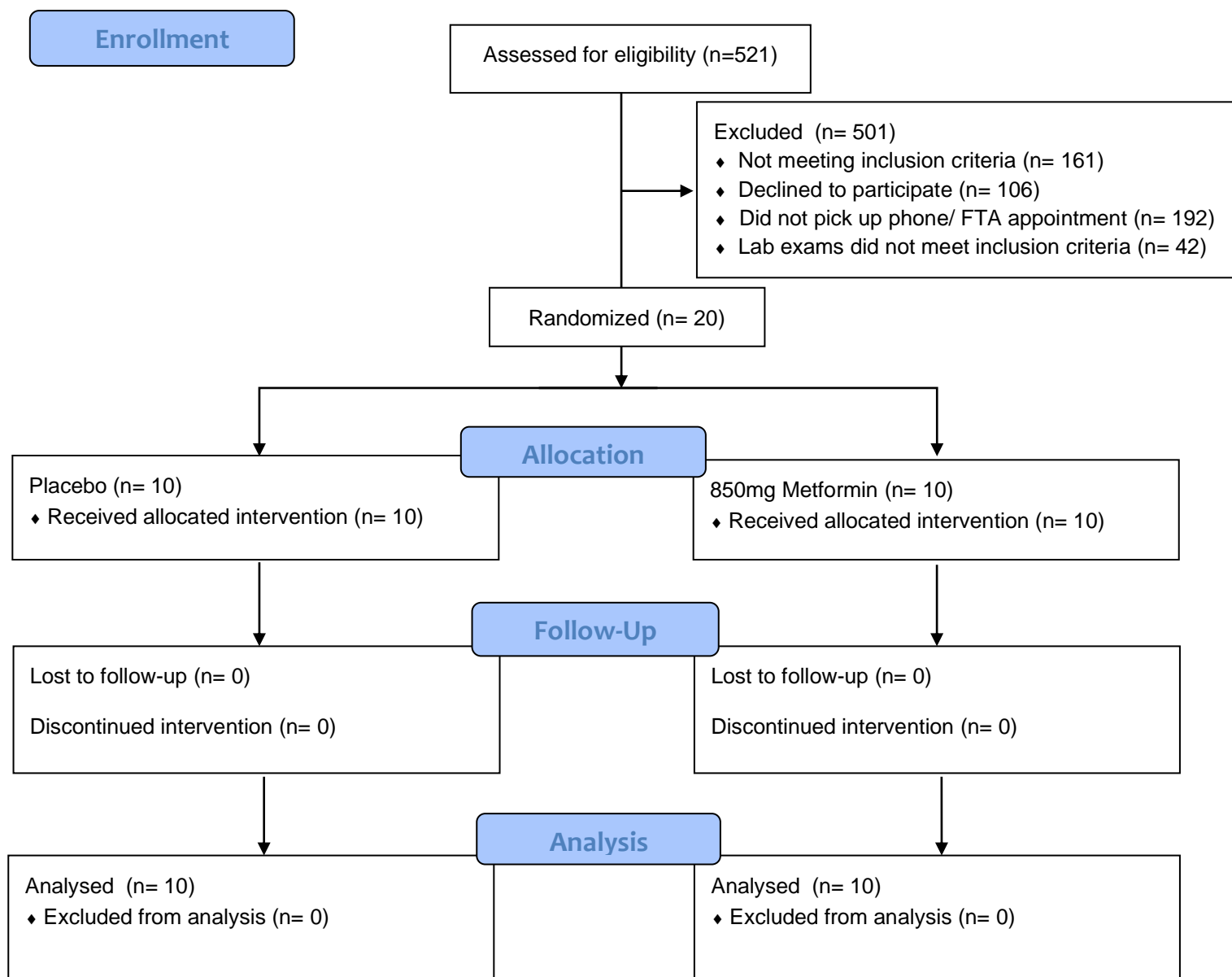

Supplement: Supplementary file 8 — Additional file 8: Figure S1. Flow chart of the study design according to CONSORT guidelines. [file 12967_2023_4456_MOESM8_ESM.pdf]

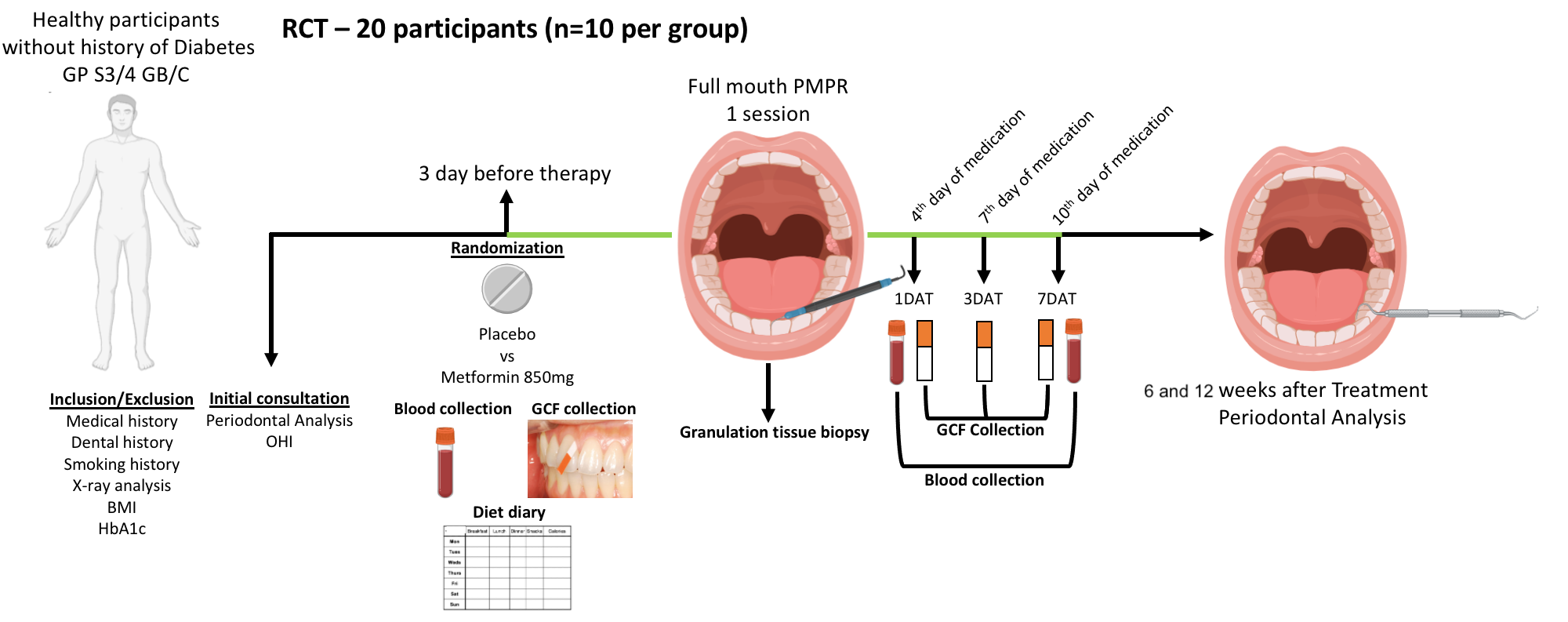

Supplement: Supplementary file 9 — Additional file 9: Figure S2. Trial design flow diagram. [file 12967_2023_4456_MOESM9_ESM.png]

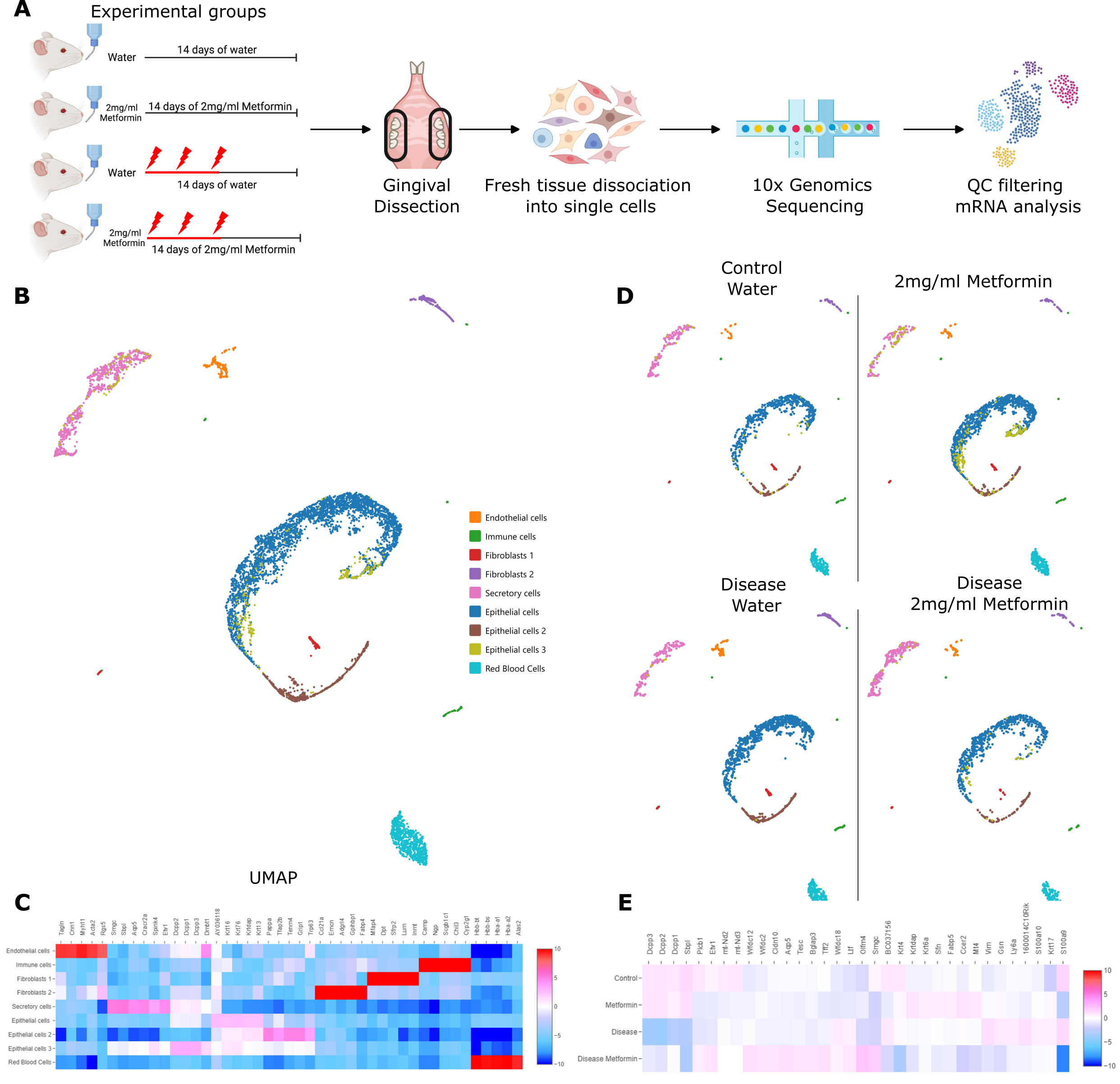

Supplement: Supplementary file 10 — Additional file 10: Figure S3. Single cell clustering of mouse gingiva in health and disease induction. [file 12967_2023_4456_MOESM10_ESM.png]

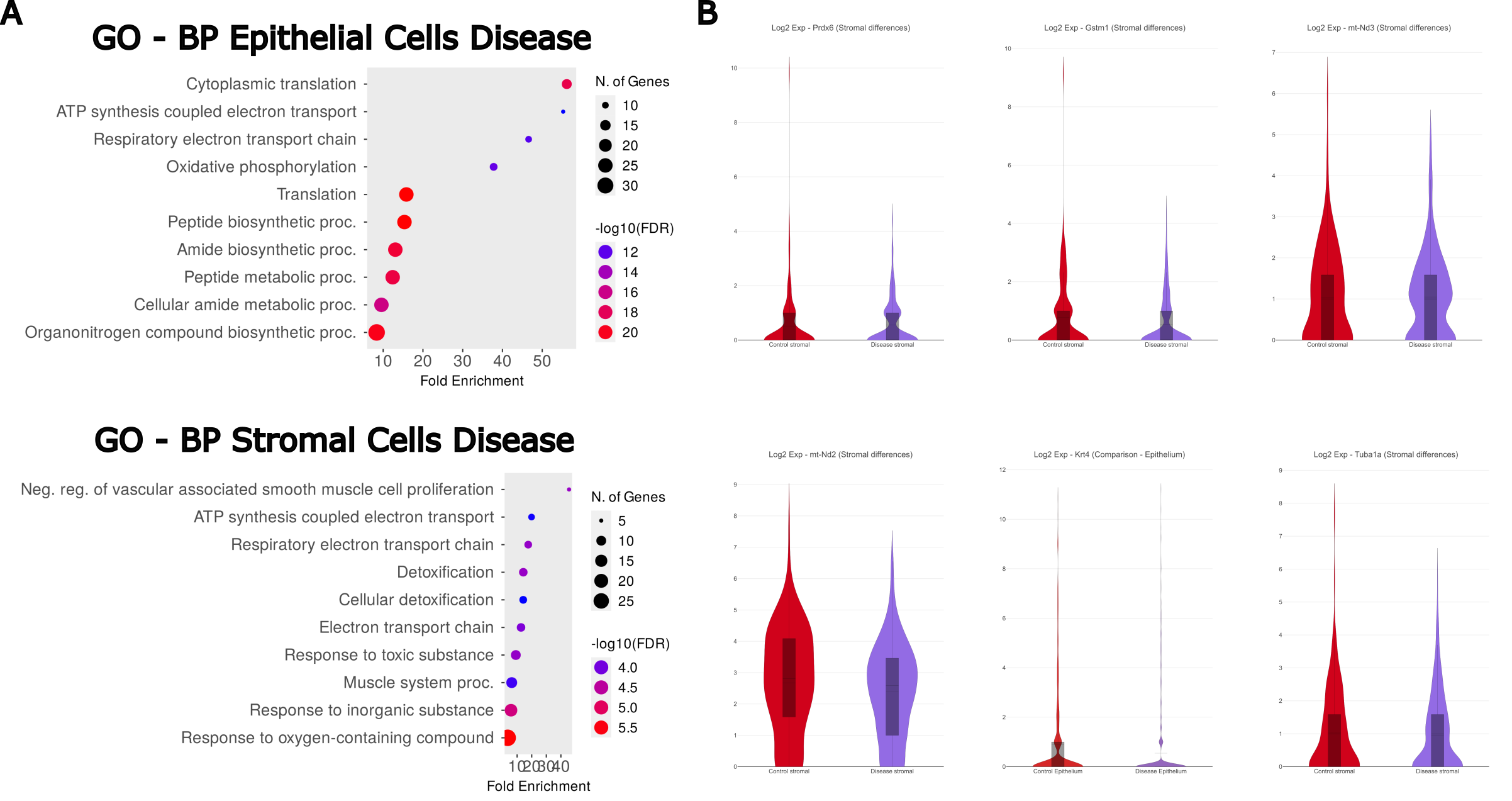

Supplement: Supplementary file 11 — Additional file 11: Figure S4. Downregulated transcriptome on epithelial and stromal compartment during disease initiation. [file 12967_2023_4456_MOESM11_ESM.png]

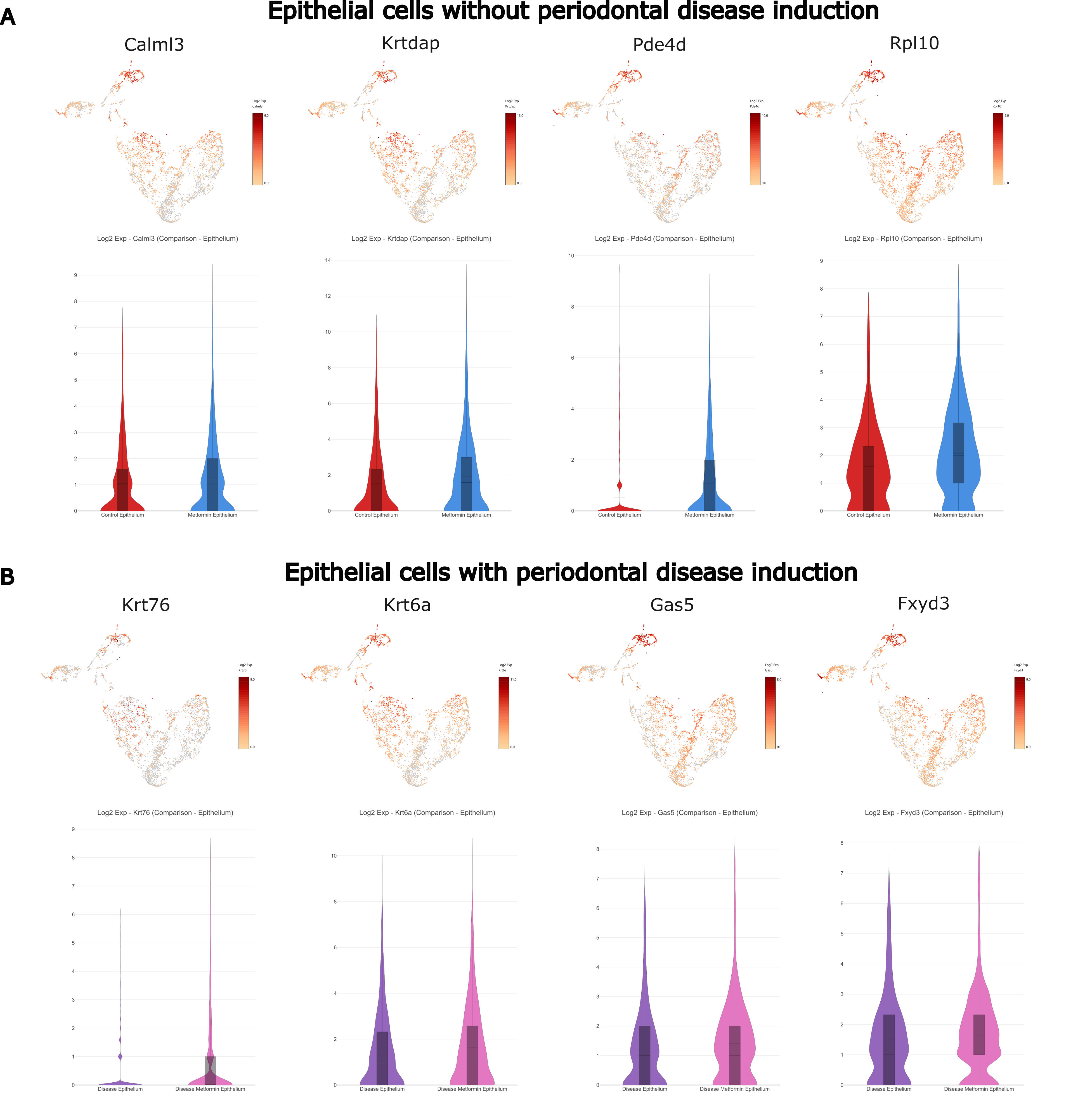

Supplement: Supplementary file 12 — Additional file 12: Figure S5. UMAPs feature and violin plots mapping the significant upregulated expression code in the Epithelial compartment when Metformin is used during homeostasis and during early disease development. [file 12967_2023_4456_MOESM12_ESM.png]

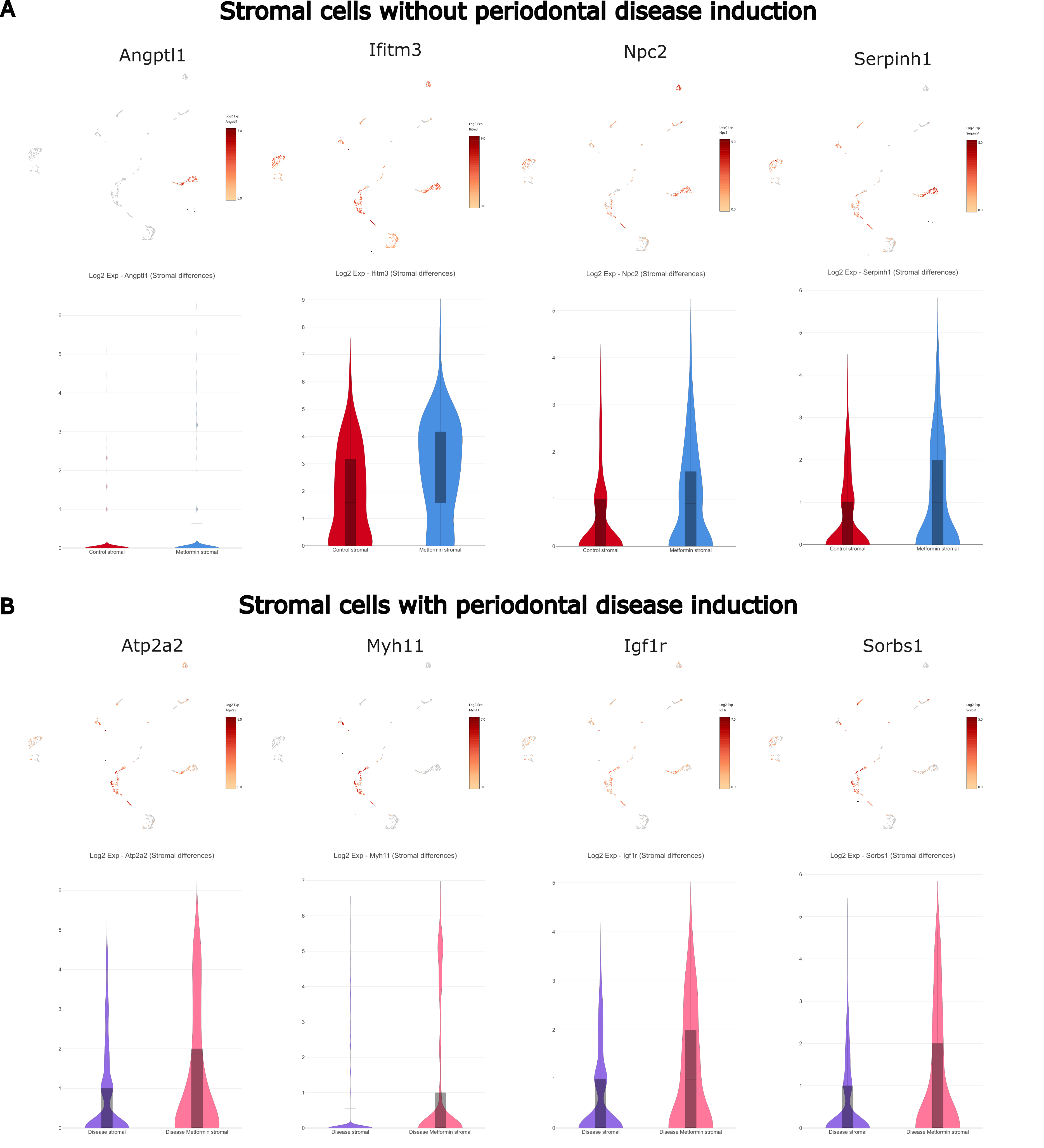

Supplement: Supplementary file 13 — Additional file 13: Figure S6. UMAPs feature and violin plots mapping the significant upregulated expression code in the Stromal compartment when Metformin is used during homeostasis and during early disease development. [file 12967_2023_4456_MOESM13_ESM.png]

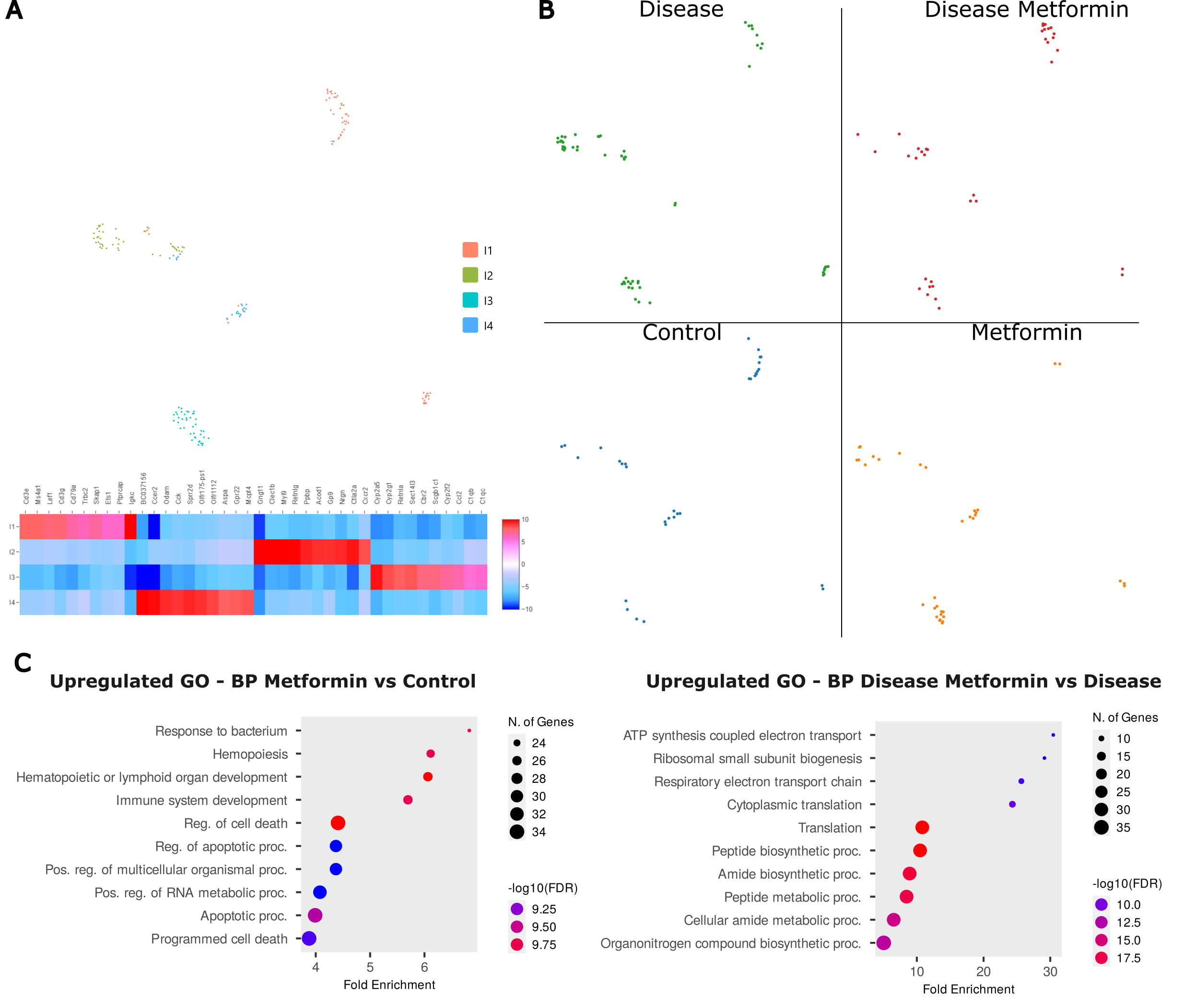

Supplement: Supplementary file 14 — Additional file 14: Figure S7. Sub analysis of immune cells clusters. [file 12967_2023_4456_MOESM14_ESM.png]

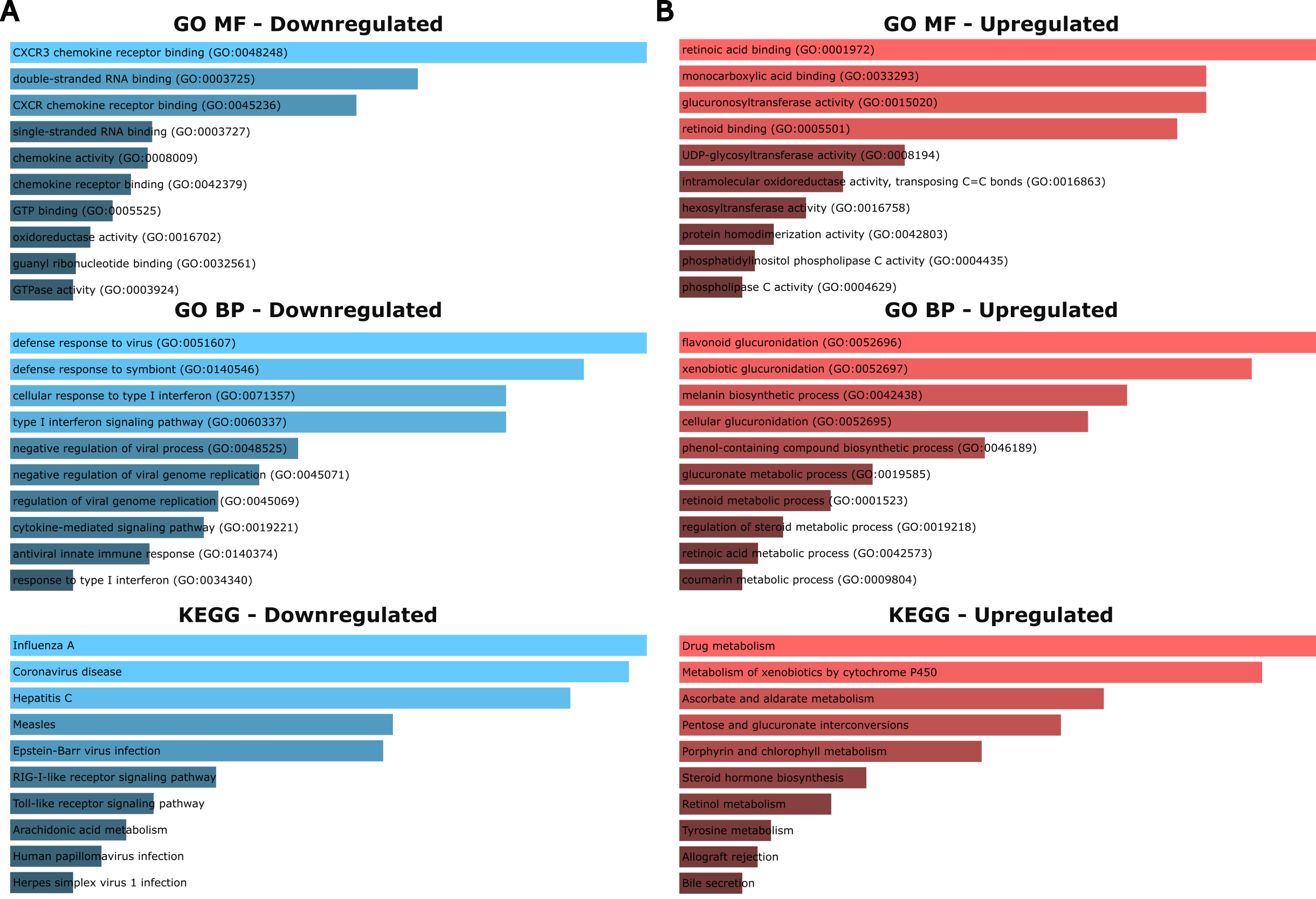

Supplement: Supplementary file 15 — Additional file 15: Figure S8. Enrichr bar plots depicting GO enrichment terms and KEGG pathways for downregulated (Blue) and Upregulated (Red) genes. [file 12967_2023_4456_MOESM15_ESM.png]
