# Supplementary material for: Repurposing Metformin for periodontal disease management as a form of oral-systemic preventive medicine
Source: J Transl Med. 2023 Oct 10;21:655. doi: 10.1186/s12967-023-04456-1 (PMC10563330; doi:10.1186/s12967-023-04456-1)
Supplement: Supplementary file 16 — Additional file 16: Table S1. Significantly expressed genes mentioned on text with P-values. Table S2. Inclusion criteria with periodontal and systemic parameters of both study groups. Table S3. Adverse effects reporting during the 10 days of medication of both study groups. Table S4. Systemic blood markers at baseline, 1 day after treatment (4 days of medication) and 7 days after treatment (10 days of medication). Table S5. Food diary evaluation during the 10 days of medication. Table S6. Up and Downregulated genes related to top enriched GO of bulk RNAseq. Table S7. GCF Luminex statistics. [file 12967_2023_4456_MOESM16_ESM.docx]

**Supplementary Tables**

|  | Gene | p-value |
| --- | --- | --- |
| Healthy Metformin vs Healthy Water | Calml3 | 0.0436939 |
|  | Pde4d | 9.31E-10 |
|  | Krtdap | 9.31E-10 |
|  | Rpl10 | 2.21E-05 |
|  | | |
| Metformin Prevention and PD vs Water and PD | Kr76 | 2.98E-19 |
|  | Kr6a | 0.0044796 |
|  | Gas5 | 0.0050556 |
|  | Foxyd3 | 0.0243145 |
|  | | |
| **Stromal cells** | | |
| Healthy Metformin vs Healthy Water | Angptl1 | 0.0014521 |
|  | Ifitm3 | 0.0084958 |
|  | Ncp2 | 0.0119806 |
|  | Serpinh1 | 0.0119806 |
|  | | |
| Metformin Prevention and PD vs Water and PD | Atp2a2 | 0.0029898 |
|  | Myh11 | 0.0001085 |
|  | Igf1r | 0.0011473 |
|  | Sorbs1 | 1.91E-06 |

**Table S1. Significantly expressed genes mentioned on text with P-values**

|  |  | Placebo | 850mg Metformin |  |
| --- | --- | --- | --- | --- |
| Variable |  | Mean (SD)/N(%) | Mean  (SD)/N(%) | p-value |
|  |  |  |  |  |
| Age (years) |  | 51.6 ± 7.5 | 54.1 ± 7.1 | 0.3229^1^ |
|  |  |  |  |  |
| Gender | *Males* | 7 (70%) | 6 (60%) | 0.26^2^ |
|  | *Females* | 3 (30%) | 4 (40%) |  |
|  |  |  |  |  |
| Periodontitis stage and grade (EFP/AAP) | *Stage III Grade B* | 4 (40%) | 4 (40%) | 0.598^2^ |
|  | *Stage IV Grade B* | 6 (60%) | 6 (60%) |  |
|  |  |  |  |  |
| BMI (<18 underweight / >30 obese) |  | 25.32 ± 3.06 | 24.93 ± 3.14 | 0.6305^1^ |
|  |  |  |  |  |
| HbA1c (>6.5%: Diabetes) |  | 5.43 ± 0.27 | 5.23 ± 0.21 | 0.0851^1^ |
|  |  |  |  |  |
| Glucose (70 a  99 mg/dL: normal range) |  | 90.5 ± 8.15 | 86.5 ± 5.85 | 0.228^1^ |
|  |  |  |  |  |
| Basal Insulin (2,6 a 24,9 µUI/mL: normal range) |  | 12.63 ± 6.29 | 13.17 ± 10.94 | 0.7477^1^ |
|  |  |  |  |  |
| Abbreviations: BMI, Body Mass Index; HbA1c, Haemoglobin A1c; SD, Standard Deviation. | | | | |

**Table S2. Inclusion criteria with periodontal and systemic parameters of both study groups.**

(1-Unpaired T-test; 2- Chi-Square Test)

|  | Placebo | 850mg Metformin |
| --- | --- | --- |
| No adverse effects | 8 (80%) | 8 (80%) |
| Adverse effects | 2 (20%) | 2 (20%) |
|  |  |  |
| Reported adverse effects | |  |
| Headache | 1 | 1 |
| Mild diarrhoea (1-2days) | 1 | 2 |
| Increase of urination | - | 1 |
| Dizziness | 1 | 1 |

**Table S3. Adverse effects reporting during the 10 days of medication of both study groups.**

| Variable | Group | Median (Min value; Max value) | | | p-value Intragroup |
| --- | --- | --- | --- | --- | --- |
|  |  | Baseline | 1 day after treatment | 7 days after treatment |  |
| Fasting Insulin  2.6-24.9µUI/mL | Placebo | 12.50 (5.40; 25.70) | 9.65 (5.30; 26.20) | 10.85 (4.70; 2.30) | 0.9761 |
|  | 850mg Metformin | 9.25 (2.30; 33.80) | 9.20 (1.50; 31.20) | 7.79 (1.70; 15.90) | ***0.0187*** |
| p-value intergroup | | 0.7055 | 0.7133 | 0.1661 |  |
| Fasting glucose  70-99mg/dL | Placebo | 89.00 (82.00; 105.00) | 88.50 (74.00; 103.00) | 89.50 (80.00; 101.00) | 0.4698 |
|  | 850mg Metformin | 84.50 (80.00; 98.00) | 85.00 (80.00; 102.00) | 84.00 (79.00; 100.00) | 0.4698 |
| p-value intergroup |  | 0.2730 | 0.2703 | 0.1509 |  |
| RBC  4.0-5.2mil/mm³ | Placebo | 5.01 (3.83; 5.35) | 4.93 (4.09; 5.5) | 5.04 (3.85; 55) | 0.5092 |
|  | 850mg Metformin | 4.59 (4.13; 5.28) | 4.48 (4.14; 5.24) | 4.43 (4.19; 5.24) | 0.3012 |
| p-value intergroup | | 0.3847 | 0.1988 | 0.0640 |  |
| Haemoglobin  12.0-16.0g/dL | Placebo | 14.70 (12.40; 15.90) | 14.70 (12.90; 15.70) | 14.70 (12.40; 15.50) | 0.2725 |
|  | 850mg Metformin | 13.45 (12.00; 16.20) | 13.40 (12.40; 16.20) | 13.30 (12.00; 16.00) | 0.4966 |
| p-value intergroup | | 0.2265 | 0.0821 | 0.0588 |  |
| Haematocrit  36.0-46.0% | Placebo | 43.85 (36.9; 46.3) | 43.15 (39.6; 46.1) | 43.85 (37.2; 46.4) | 0.6703 |
|  | 850mg Metformin | 41.75 (37.8; 48.8) | 41.35 (36.4; 49.1) | 40.5 (37.7; 48.8) | 0.6219 |
| p-value intergroup | | 0.4497 | 0.1620 | 0.1041 |  |
| MCV  80.0-100.0µ³ | Placebo | 87.95 (84.30; 96.30) | 87.80 (83.80; 96.80) | 87.70 (84.40; 96.60) | 0.8395 |
|  | 850mg Metformin | 89.00 (82.00; 97.60) | 88.25 (85.20; 98.60) | 88.80 (82.40; 99.00) | 0.3413 |
| p-value intergroup | | 0.6501 | 0.4274 | 0.6232 |  |
| MCH  26.0-34.0pg | Placebo | 29.80 (27.30; 32.40) | 29.75 (27.30; 32.30) | 29.70 (26.90; 32.20) | 0.6219 |
|  | 850mg Metformin | 29.35 (26.70; 32.20) | 29.55 (27.20; 32.30) | 29.50 (26.60; 32.70) | 0.4066 |
| p-value intergroup | | 0.7055 | 0.7337 | 0.6501 |  |
| MCHC  31.0-37.0g/dl | Placebo | 33.50 (32.40; 34.60) | 33.45 (32.50; 35.10) | 33.35 (31.90; 35.40) | 0.9048 |
|  | 850mg Metformin | 33.00 (31.70; 33.90) | 32.75 (31.60; 34.30) | 32.90 (31.80; 33.80) | 0.9277 |
| p-value intergroup | | 0.0539 | 0.0696 | 0.1212 |  |
| RDW  10.0-15.0% | Placebo | 12.65 (11.70; 13.50) | 12.60 (11.50; 13.00) | 12.55 (11.40; 13.20) | 0.1813 |
|  | 850mg Metformin | 12.65 (11.30; 13.50) | 12.65 (11.30; 13.00) | 12.65 (11.40; 12.80) | 0.4966 |
| p-value intergroup | | 0.9397 | 0.7913 | 0.8501 |  |
| Leucocytes  4.500-11.000mm3 | Placebo | 6155 (5590; 12130) | 6340 (5280; 9510) | 6725 (5210; 9090) | 0.8395 |
|  | 850mg Metformin | 6645 (4720; 10180) | 5975 (4330; 9620) | 6370 (5000; 10270) | 0.3012 |
| p-value intergroup | | 0.8798 | 0.8798 | 0.8206 |  |
| Neutrophils  1.800-7.000mm3 | Placebo | 3782.70 (2828.50; 9934.40) | 3820.40 (3056.90; 7237.10) | 3718.20 (2784.90; 6626.70) | 0.4966 |
|  | 850mg Metformin | 3845.45 (2458.20; 5955.30) | 3821.10 (2740.80; 7420.30) | 3631.55 (2740.50; 6034.20) | 0.6703 |
| p-value intergroup | | 1.0000 | 0.9397 | 0.7055 |  |
| Eosinophils  0-600mm3 | Placebo | 115.95 (68.9; 282.3) | 115.45 (38.6; 169) | 137.35 (69.7; 230.6) | 0.2019 |
|  | 850mg Metformin | 105.4 (20.8; 579.7) | 94.35 (11.2; 527.3) | 130.7 (17.7; 473) | 0.4066 |
| p-value intergroup | | 0.8206 | 0.7624 | 0.7055 |  |
| Basophils  0-200mm3 | Placebo | 44.45 (18.00; 72.89) | 37.25 (21.19; 68.00) | 36.55 (20.80; 81.80) | 0.4066 |
|  | 850mg Metformin | 30.70 (9.69; 61.49) | 37.79 (9.89; 51.80) | 41.95 (11.10; 71.90) | ***0.0247*** |
| p-value intergroup | | 0.4057 | 0.5967 | 0.8798 |  |
| Lymphocytes  1.200-5.000mm3 | Placebo | 1812.00 (1334.30; 2862.20) | 1810.40 (1241.20; 2711.00) | 2024.50 (1240.00; 2947.10) | 0.2725 |
|  | 850mg Metformin | 1903.90 (929.80; 2942.00) | 1660.05 (1047.90; 2501.20) | 1914.00 (1070.00; 3440.50) | ***0.0136*** |
| p-value intergroup | | 0.6501 | 0.5967 | 0.9397 |  |
| Monocytes  80-1.200mm3 | Placebo | 491.55 (332.30; 710.20) | 430.60 (321.00; 798.70) | 507.25 (349.10; 944.80) | 0.9048 |
|  | 850mg Metformin | 420.00 (358.70; 855.10) | 494.65 (349.70; 740.70) | 452.00 (390.10; 613.60) | 0.9048 |
| p-value intergroup | | 0.3643 | 0.7624 | 0.5454 |  |
| Platelets  150-400mil/mm³ | Placebo | 231.00 (196.00; 417.00) | 222.50 (183.00; 376.00) | 258.00 (206.00; 407.00) | 0.3413 |
|  | 850mg Metformin | 268.50 (200.00; 323.00) | 270.00 (185.00; 350.00) | 292.00 (176.00; 314.00) | 0.4966 |
| p-value intergroup | | 0.7624 | 0.7624 | 0.7055 |  |
| TNF-a  >8.1pg/mL | Placebo | 6.90 (4.00; 8.00) | 6.60 (4.00; 8.00) | 6.75 (4.00; 9.60) | 0.3114 |
|  | 850mg Metformin | 7.30 (4.00; 9.20) | 7.40 (4.00; 9.90) | 7.00 (4.00; 10.30) | 0.0930 |
| p-value intergroup | | 0.9646 | 0.5676 | 0.6304 |  |
| ESR  <10mm in the first hour | Placebo | 5 (2; 12) | 4 (2; 20) | 6 (2; 24) | ***0.0460*** |
|  | 850mg Metformin | 5 (3; 25) | 6 (3; 45) | 7 (2; 35) | 0.7471 |
| p-value intergroup | | 0.1155 | 0.2746 | 0.3314 |  |
| Fibrinogen  146-400mg/dL | Placebo | 268 (194; 349) | 286 (224; 369) | 281 (216; 352) | 0.8438 |
|  | 850mg Metformin | 259 (190; 395) | 281 (234; 372) | 281 (207; 352) | 0.3508 |
| p-value intergroup | | 0.2968 | 0.3985 | 0.4296 |  |
| hsCRP  <5.0mg/L | Placebo | 1.49 (0.50; 3.60) | 2.13 (0.60; 16.83) | 1.91 (0.50; 9.61) | ***0.0482*** |
|  | 850mg Metformin | 1.04 (0.60; 3.07) | 2.01 (0.60; 4.38) | 1.1 (0.29; 3.80) | 0.3675 |
| p-value intergroup | | 0.2118 | 0.3089 | 0.1529 |  |
| IL-6  <7.0pg/mL | Placebo | 2.7 (1.5; 4.7) | 3.6 (1.5; 6.7) | 3.45 (1.5; 5.6) | 0.1641 |
|  | 850mg Metformin | 2.35 (1.5; 3.8) | 3.1 (1.5; 6.6) | 2.3 (1.5; 8.4) | 0.1040 |
| p-value intergroup | | 0.1595 | 0.3692 | 0.3350 |  |

**Table S4. Systemic blood markers at baseline, 1 day after treatment (4 days of medication) and 7 days after treatment (10 days of medication).**

Bold and Italic values indicate statistical significance (Intergroup test: Mann Whitney; Intragroup test: Friedman test).

|  | Placebo  Mean (%)/ (SD) | 850mg Metformin  Mean (%)/ (SD) | p-values |
| --- | --- | --- | --- |
| Overall number of servings in 10 days | 93.30 ± 14.80 | 97.40 ± 28.94 | 0.6948 |
| Potatoes, bread, rice, pasta and other starchy carbohydrate foods | 33.37 ± 5.92 | 30.23 ± 5.41 | 0.2319 |
| Fruit and vegetables | 13.22 ± 7.58 | 14.99 ± 8.90 | 0.6377 |
| Dairy and alternatives | 6.19 ± 5.51 | 6.46 ± 4.59 | 0.9068 |
| Beans, pulses, fish, eggs, meat and other protein | 29.40 ± 8.23 | 23.20 ± 10.00 | 0.1477 |
| Oils and spreads | 0.87 ± 1.76 | 1.19 ± 2.55 | 0.7467 |
| Processed foods high in fat, salt, and/or sugar | 8.01± 5.88 | 13.75 ± 5.53 | **0.0375** |
| Coffee and tea | 8.89 ± 3.94 | 10.14 ± 6.86 | 0.6218 |

**Table S5. Food diary evaluation during the 10 days of medication.**

Food grouping based on the NHS Eatwell guide. Note: Bold values indicate statistical significance. Significant p-values (p < .05) are provided in italics. (Unpaired T-Test).

| Upregulated | |  |
| --- | --- | --- |
| ID | **Name** | **Protein function** |
| HMGCS2 | 3-hydroxy-3-methylglutaryl-CoA synthase 2 | Catalyses the first reaction of ketogenesis |
| UGT1A1 | UDP glucuronosyltransferase family 1 member A1 | Glucuronidation |
| UGT1A6 | UDP glucuronosyltransferase family 1 member A6 | Glucuronidation |
| UGT1A8 | UDP glucuronosyltransferase family 1 member A8 | Glucuronidation |
| UGT1A9 | UDP glucuronosyltransferase family 1 member A9 | Glucuronidation |
| CKMT1B | creatine kinase, mitochondrial 1B | Transfer of high energy phosphate |
| GSTA1 | glutathione S-transferase alpha 1(GSTA1) | Detoxification and protection from ROS |
| PRODH | proline dehydrogenase 1 | Catalyses the first step in proline degradation |
|  |  |  |
| Downregulated | |  |
| ID | **Name** | **Protein function** |
| OAS2 | 2'-5'-oligoadenylate synthetase 2 | Innate immune response to viral infection |
| CXCL10 | C-X-C motif chemokine ligand 10 | Inflammatory chemokine |
| MX1 | MX dynamin like GTPase 1(MX1) | Interferon mediated cellular antiviral response |
| MX2 | MX dynamin like GTPase 1(MX2) | Interferon mediated cellular antiviral response |
| EIF2AK2 | eukaryotic translation initiation factor 2 alpha kinase 2 | Innate immune response to viral infection |
| HLA-DMA | major histocompatibility complex, class II, DM alpha | Antigen presenting molecule |
| RSAD2 | radical S-adenosyl methionine domain containing 2 | Interferon-inducible antiviral response |
| STAT1 | signal transducer and activator of transcription 1 | Interferon mediated response to pathogen |

**Table S6. Up and Downregulated genes related to top enriched GO of bulk RNAseq.**

| Variable  (pg/ml) | Group | Baseline | 1 day after treatment | 3 days after treatment | 7 days after treatment | p-value intragroup |
| --- | --- | --- | --- | --- | --- | --- |
|  |  | Mean (SD) | Mean (SD) | Mean (SD) | Mean (SD) |  |
| Insulin | Placebo | 18.99 (8.53) | 46.78 (28.21) | 25.03 (15.47) | 19.76 (12.03) | ***0.0188*** |
|  | 850mg Metformin | 17.25 (10.54) | 64.69 (52.63) | 39.42 (38.34) | 36.81 (45.88) | ***0.039*** |
|  | p-value between groups | 0.3452 | 0.1777 | 0.1427 | 0.1354 |  |
|  |  |  |  |  |  |  |
| Leptin | Placebo | 101.20 (21.39) | 133.47 (53.32) | 109.22 (29.85) | 102.41 (27.32) | 0.0582 |
|  | 850mg Metformin | 87.29 (18.10) | 156.07 (59.59) | 97.52 (31.11) | 96.51 (30.56) | ***0.0074*** |
|  | p-value between groups | 0.067 | 0.1903 | 0.201 | 0.3274 |  |
|  |  |  |  |  |  |  |
| CRP | Placebo | 165.20 (228.30) | 3058.48 (5441.50) | 1378.21 (2857.19) | 306.59 (460.10) | 0.1718 |
|  | 850mg Metformin | 97.8 (157.95) | 4665.98 (7435.42) | 206.90 (306.65) | 1553.08 (3377.95) | 0.1136 |
|  | p-value between groups | 0.2263 | 0.294 | 0.1069 | 0.2627 |  |
|  |  |  |  |  |  |  |
| IL-6 | Placebo | 3.78 (1.29) | 36.49 (27.26) | 13.91 (12.40) | 3.79 (1.96) | ***0.0036*** |
|  | 850mg Metformin | 3.62 (1.33) | 115.47 (124.20) | 10.44 (10.60) | 7.64 (10.00) | ***0.0174*** |
|  | p-value between groups | 0.3989 | ***0.0326*** | 0.2549 | 0.1241 |  |
|  |  |  |  |  |  |  |
| VEGF | Placebo | 67.42 (16.72) | 69.09 (36.85) | 67.49 (34.80) | 54.30 (38.19) | 0.2689 |
|  | 850mg Metformin | 50.79 (26.27) | 84.60 (68.12) | 73.92 (64.91) | 57.68 (34.21) | 0.1567 |
|  | p-value between groups | ***0.0451*** | 0.2672 | 0.3928 | 0.4185 |  |
|  |  |  |  |  |  |  |
| Angiogenin | Placebo | 392.11 (243.82) | 770.06 (390.61) | 409.40 (220.41) | 276.11 (97.76) | ***0.0038*** |
|  | 850mg Metformin | 364.11 (238.81) | 1198.04 (568.95) | 393.94 (223.76) | 393.14 (202.34) | ***0.0002*** |
|  | p-value between groups | 0.3991 | ***0.0328*** | 0.439 | 0.0585 |  |
|  |  |  |  |  |  |  |
| IL-10 | Placebo | 0.76 (0.43) | 0.72 (0.36) | 0.41 (0.19) | 0.35 (0.18) | ***0.0262*** |
|  | 850mg Metformin | 0.79 (0.54) | 0.66 (0.46) | 0.47 (0.20) | 0.55 (0.40) | 0.3019 |
|  | p-value between groups | 0.4449 | 0.3842 | 0.2341 | 0.0904 |  |
|  |  |  |  |  |  |  |
| Vitamin D | Placebo | 72201.82 (84678.91) | 17456.20 (9763.79) | 28108.50 (32488.05) | 16171.57 (11054.01) | 0.0553 |
|  | 850mg Metformin | 46997.36 (70117.21) | 48580.92 (51288.38) | 32829.01 (64695.23) | 14953.52 (15785.78) | 0.0838 |
|  | p-value between groups | 0.2389 | ***0.0376*** | 0.4195 | 0.4205 |  |

**Table S7. GCF Luminex statistics.**

Note: Bold values indicate statistical significance. Significant p-values (p < .05) are provided in italics.

Intergroup: Unpaired T-test

Intragroup: Repeated Measures one-way ANOVAss
